# Eudesmane-Type Sesquiterpene Glycosides from *Dictamnus dasycarpus* Turcz.

**DOI:** 10.3390/molecules23030642

**Published:** 2018-03-13

**Authors:** Shengcai Yang, Zheng Li, Jianli Wang, Jingya Ruan, Chang Zheng, Peijian Huang, Lifeng Han, Yi Zhang, Tao Wang

**Affiliations:** 1 Tianjin State Key Laboratory of Modern Chinese Medicine, 312 Anshanxi Road, Nankai District, Tianjin 300193, China; 15122473723@163.com (S.Y.); wo15510977612@163.com (Z.L.); Ruanjy19930919@163.com (J.R.); 2 Tianjin Key Laboratory of TCM Chemistry and Analysis, Institute of Traditional Chinese Medicine, Tianjin University of Traditional Chinese Medicine, 312 Anshanxi Road, Nankai District, Tianjin 300193, China; wjl15802226160@126.com (J.W.); 18702270347@163.com (C.Z.); hpjforever@sina.com (P.H.); hanlifeng_1@sohu.com (L.H.)

**Keywords:** *Dictamnusdasycarpus* Turcz., sesquiterpene glycoside, eudesmane-type sesquiterpene, dictameudesmnoside, triglyceride accumulation, HepG2 cell

## Abstract

Eudesmane-type sesquiterpenes have been reported to exhibit varieties of biological activities. During the process of investigating this kind of natural product from the root bark of *Dictamnus dasycarpus* Turcz., 13 eudesmane-type sesquiterpene glycosides including six new isolates, named as dictameudesmnosides A_1_ (**1**), A_2_ (**2**), B (**3**), C (**4**), D (**5**), and E (**6**), together with seven known ones (**7**–**13**), were obtained. Herein, their structures were determined by the analysis of physical data, spectroscopic analysis, and chemical methods. The existence of α-configuration glucose units in their structures (**1**–**5**, **8**) is not very common in natural glycosidic components. Meanwhile, compounds **3**–**5**, **7**, and **9**–**13** displayed TG accumulation inhibitory effects on HepG2 cells.

## 1. Introduction

Eudesmane-type sesquiterpenes and their glycosides, as one of the secondary metabolites, show broad bioactivities, including anti-inflammatory [1], anticancer [2,3], anti-angiogenic [4], antifungal [5], and anti-hepatitis B virus [6] activities, which make phytochemical or bioactive investigations into these kinds of constituents meaningful.

The root bark of *Dictamnus dasycarpus* Turcz., also known as Cortex Dictamni, belongs to the Rutaceae family. It is a plant rich in eudesmane-type sesquiterpenes [7,8]. As a traditional Chinese medicine, *D. dasycarpus* has been commonly used in the treatment of inflammation, microbial infection, pruritus vulvae, eczema, scabies, cancer, and other diseases in China for thousands of years because of its abilities to arrest itching, clear away heat, and eliminate dampness [9]. In addition, the decoction could effectively inhibit body weight and fat content increase caused by high fat diet, reduce triglyceride (TG) and total cholesterol contents in blood, and alleviate obesity [10].

During the process of investigating eudesmane-type sesquiterpenes from the root bark of *D. dasycarpus*, 13 compounds including six new isolates, named as dictameudesmnosides A_1_ (**1**), A_2_ (**2**), B (**3**), C (**4**), D (**5**), and E (**6**), together with seven known ones, dictamnosides B (**7**) [7], G (**8**) [11], C (**9**) [7], A (**10**) [7], L (**11**) [8], D (**12**) [7], and K (**13**) [8], were obtained. Meanwhile, *in vitro* bioactivity screening experiments testing the TG inhibitory effect for the 13 compounds were performed. We herein report the isolation and characterization of the abovementioned constituents along with their bioassay results.

## 2. Results and Discussion

**Figure 1:**
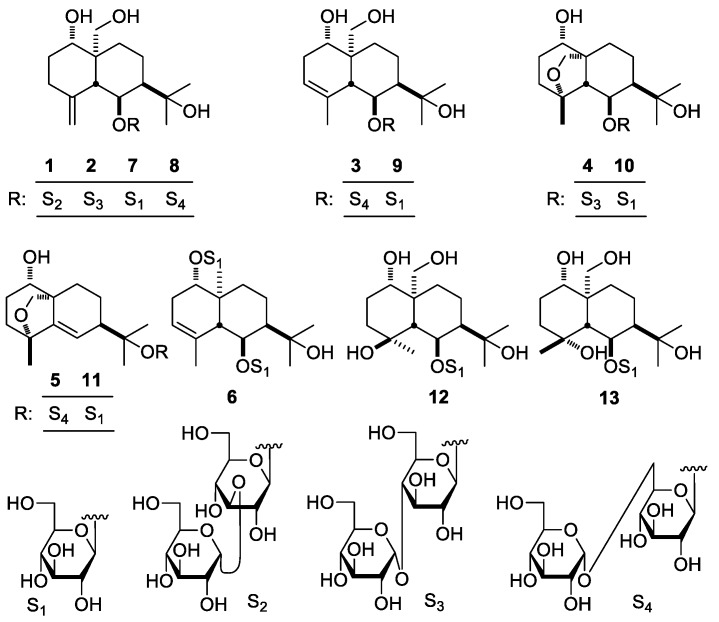
Compounds **1**–**13** obtained from *D. dasycarpus*.

The 70% ethanol-water extract from *D. dasycarpus* root bark was partitioned in an EtOAc–H_2_O mixture to afford EtOAc and H_2_O layer extract, respectively. The H_2_O layer extract was subjected to D101 macroporous resin column chromatography (CC) and eluted with H_2_O and 95% EtOH, successively. Then, 95% EtOH eluted fraction was isolated by silica gel, Sephadex LH-20 CC, and preparative high-performance liquid chromatography (pHPLC) to yield compounds **1**–**13** (Figure 1).

**Figure 2:**
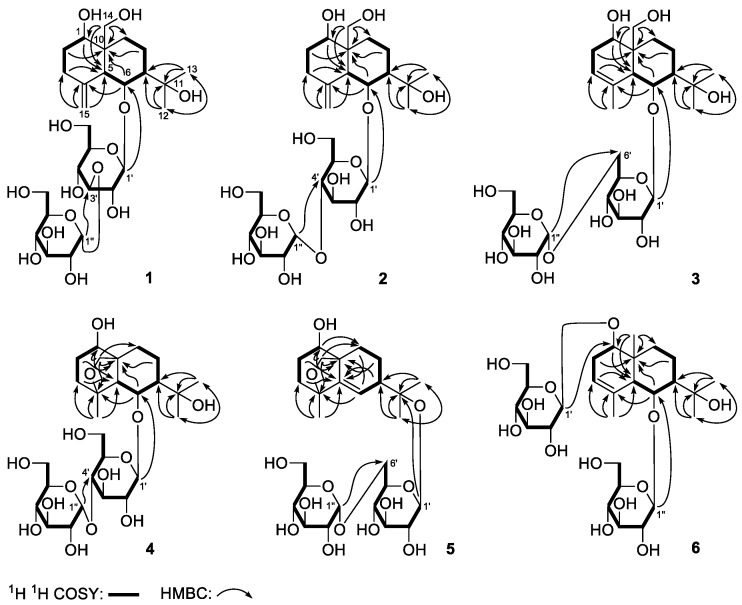
The main ^1^H–^1^H COSY, and HMBC correlations of **1**–**6**.

**Figure 3:**
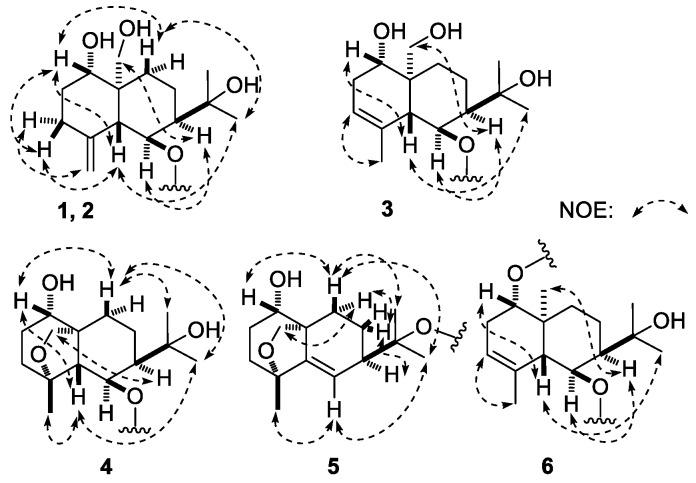
The main NOE correlations of the aglycons of **1**–**6**.

Dictameudesmnoside A_1_ (**1**) was isolated as white powder with positive optical rotation, [α${]}_{D}^{25}$ + 53.5 (MeOH). Its molecular formula was deduced to be C_27_H_46_O_14_ by the negative-ion HRESI-TOF-MS analysis (*m/z* 593.2826 [M − H]^−^; calcd for C_27_H_45_O_14_, 593.2815). Acid hydrolysis of **1** yielded d-glucose, which was identified by its retention time and optical rotation using chiral detection by HPLC analysis [12]. The ^1^H and ^13^C-NMR (Table 1, CD_3_OD) spectra (Supplementary data) of **1**, which were assigned by various 2D-NMR experiments (^1^H–^1^H COSY, HSQC, HMBC), showed signals assignable to one β-d-glucopyranosyl (δ 4.47 (1H, d, *J* = 8.0 Hz, H-1′)), and one α-d-glucopyranosyl (δ 5.12 (1H, d, *J* = 3.5 Hz, H-1″)). Twenty-seven carbon signals were displayed in its ^13^C-NMR spectrum. In addition to the carbon signals represented by β-d-glucopyranosyl and α-d-glucopyranosyl, the other 15 signals indicated that the aglycon of **1** was an eudesmane-type sesquiterpene, consisting of two tertiary methyl groups (δ 1.31, 1.33 (3H each, both s, H_3_-12, 13)), one oxygenated methylene group (δ 3.45, 3.88 (1H each, both d, *J* = 11.5 Hz, H_2_-14)), two oxygenated methine protons (δ 3.60 (1H, m, overlapped, H-1), 4.51 (1H, dd, *J* = 5.0, 6.5 Hz, H-6)), together with one terminal olefinic moiety (δ 4.88, 4.92 (1H each, both br. s, H_2_-15)). The ^1^H–^1^H COSY spectrum of **1** suggested the presence of four partial structures written in bold lines, as shown in Figure 2. Its planar structure was determined based on the key HMBC correlations from the following proton and carbon pairs: δ_H_ 3.60 (H-1) to δ_C_ 51.8 (C-5); δ_H_ 1.75, 1.94 (H_2_-2) to δ_C_ 43.5 (C-10); δ_H_ 2.22, 2.33 (H_2_-3) to δ_C_ 51.8 (C-5); δ_H_ 1.62, 1.83 (H_2_-8), 4.51 (H-6) to δ_C_ 43.5 (C-10); δ_H_ 1.31 (H_3_-12) to δ_C_ 28.7 (C-13), 46.7 (C-7), 74.3 (C-11); δ_H_ 1.33 (H_3_-13) to δ_C_ 30.6 (C-12), 46.7 (C-7), 74.3 (C-11); δ_H_ 3.45, 3.88 (H_2_-14) to δ_C_ 28.7 (C-9), 43.5 (C-10), 51.8 (C-5), 82.4 (C-1); δ_H_ 4.88, 4.92 (H_2_-15) to δ_C_ 36.8 (C-3), 51.8 (C-5), 147.7 (C-4); δ_H_ 4.47 (H-1′) to δ_C_ 78.4 (C-6); δ_H_ 5.12 (H-1″) to δ_C_ 88.0 (C-3′), which were very similar to those of dictamnoside B (**7**) [7], except for the signals due to the sugar moiety. The relative configuration was further elucidated by the NOESY experiment, and NOE correlations between δ_H_ 3.60 (H-1) and δ_H_ 1.59 (Hβ-9), 2.22 (Hβ-3), 2.55 (H-5); δ_H_ 1.98 (H-7) and δ_H_ 3.45 (H-14a), 4.51 (H-6); δ_H_ 1.31 (H_3_-12) and δ_H_ 1.59 (Hβ-9), 2.55 (H-5) (Figure 3) were observed. Consequently, the structure of dictameudesmnoside A_1_ (**1**) was formulated as 5β,7α(*H*),10α-eudesm-4(15)-ene-1α,6β,11,14-tetraol 6-*O*-α-d-glucopyranosyl(1→3)-β-d-glucopyranoside.

Dictameudesmnoside A_2_ (**2**) was obtained as a white powder with positive optical rotation ([α${]}_{D}^{25}$ +32.1, MeOH). Its HRESI-TOF-MS showed the [M + COOH]^−^ ion peak at *m/z* 639.2883 (calcd for C_28_H_47_O_16_, 639.2870), consistent with the same molecular formula, C_27_H_46_O_14_, as that of **1**. The ^1^H and ^13^C-NMR spectroscopic data comparison of **2** (Table 2, CD_3_OD) with **1** revealed that both of them have the same aglycon, 5β,7α(*H*),10α-eudesm-4(15)-ene-1α,6β,11,14-tetraol (δ 1.31, 1.32 (3H each, both s, H_3_-12, 13), 3.44, 3.87 (1H each, both d, *J* = 12.0 Hz, H_2_-14), 3.62 (1H, m, H-1), 4.50 (1H, dd, *J* = 5.0, 6.5 Hz, H-6), 4.88, 4.91 (1H each, both br. s, H_2_-15)], one β-d-glucopyranosyl [δ 4.44 (1H, d, *J* = 8.0 Hz, H-1′)], along with one α-d-glucopyranosyl [δ 5.14 (1H, d, *J* = 3.5 Hz, H-1″)). Finally, the linkage positions of the abovementioned groups were determined by the long-range correlations observed from δ_H_ 4.44 (H-1′) to δ_C_ 78.4 (C-6); δ_H_ 5.14 (H-1″) to δ_C_ 80.8 (C-4′) in the HMBC experiment. Furthermore, the NOE correlations between proton and proton pairs were similar to those of **1**. Then, the structure of dictameudesmnoside A_2_ (**2**) was established as 5β,7α(*H*),10α-eudesm-4(15)-ene-1α,6β,11,14-tetraol 6-*O*-α-d-glucopyranosyl(1→4)-β-d-glucopyranoside.

Dictameudesmnoside B (**3**) was obtained as white power as well. On the basis of the HRESI-TOF-MS mass spectra, the molecular formula of **3** was deduced to be C_27_H_46_O_14_ (*m/z* 639.2886 [M + COOH]^−^; calcd for C_28_H_47_O_16_, 639.2870), the same as that of **1** and **2**. Its ^1^H and ^13^C-NMR (Table 3, CD_3_OD) spectra (Supplementary data) denoted that there were the same sugar moieties as the abovementioned two isolates: β-d-glucopyranosyl (δ 4.60 (1H, d, *J* = 8.0 Hz, H-1′)), and α-d-glucopyranosyl (δ 4.80 (1H, d, *J* = 4.0 Hz, H-1″)). Twenty-seven carbon signals similar to compounds **1** and **2** were displayed in its ^13^C-NMR spectrum. The abovementioned evidence indicated that **3** was the isomer of **1** and **2**. The ^1^H and ^13^C-NMR spectra along with 2D-NMR, including ^1^H–^1^H COSY, HSQC, and HMBC spectra, suggested three methyl (δ 1.30, 1.33, 1.83 (3H each, all s, H_3_-12, 13, 15)), one oxygenated methylene (δ [3.70 (1H, m, overlapped), 3.96 (1H, d, *J* = 11.0 Hz)], H_2_-14), two oxygenated methine (δ 3.76 (1H, m, H-1), 4.49 (1H, dd, *J* = 3.5, 4.5 Hz, H-6)), together with one trisubstitued olefinic bond (δ 5.31 (1H, br. s, H-3)) present in its aglycon. The existences of ‘‘–O–CH–CH_2_–CH=C–’’ and ‘‘–CH–CH(O)–CH–CH_2_–CH_2_–’’ moieties were clarified by the correlations between proton and proton found in the ^1^H–^1^H COSY spectrum. Meanwhile, the planar structure of its aglycon was determined by the long-range correlations observed from δ_H_ 3.76 (H-1) to δ_C_ 51.0 (C-5); δ_H_ 2.12, 2.52 (H_2_-2) to δ_C_ 42.7 (C-10); δ_H_ 5.31 (H-3) to δ_C_ 51.0 (C-5); δ_H_ 1.83, 2.00 (H_2_-8), 4.49 (H-6) to δ_C_ 42.7 (C-10); δ_H_ 1.30 (H_3_-12)to δ_C_ 30.4 (C-13), 45.3 (C-7), 74.2 (C-11); δ_H_ 1.33 (H_3_-13) to δ_C_ 29.8 (C-12), 45.3 (C-7), 74.2 (C-11); δ_H_ 3.70, 3.96 (H_2_-14) to δ_C_ 26.6 (C-9), 42.7 (C-10), 51.0 (C-5), 79.6 (C-1); δ_H_ 1.83 (H_3_-15) to δ_C_ 51.0 (C-5), 122.3 (C-3), 135.2 (C-4) in its HMBC experiment, which was consistent with that of dictamnoside C (**9**) [7]. The relative configuration of **3** was elucidated according to the NOE correlations between δ_H_ 2.92 (H-5) and δ_H_ 1.30 (H_3_-12), 3.76 (H-1); δ_H_ 1.97 (H-7) and δ_H_ 3.70 (H-14a), 4.49 (H-6) (Figure 3) displayed in the NOESY spectrum. Then, the aglycon of dictameudesmnoside B (**3**) was elucidated to be 5β,7α(*H*),10α-eudesm-3-ene-1α,6β,11,14-tetraol. Finally, the linkage positions of the sugar moieties were determined by the long-range correlations observed from δ_H_ 4.60 (H-1′) to δ_C_ 79.4 (C-6); δ_H_ 4.80 (H-1″) to δ_C_ 68.2 (C-6′). On the basis of abovementioned evidence, the structure of dictameudesmnoside B (**3**) was deduced to be 5β,7α(*H*),10α-eudesm-3-ene-1α,6β,11,14-tetraol 6-*O*-α-d-glucopyranosyl(1→6)-β-d-glucopyranoside.

Dictameudesmnoside C (**4**) was obtained as a white power. Its molecular formula was established as C_27_H_46_O_14_ based on the [M + COOH]^−^ quasi-molecular ion at *m/z* 639.2878 (calcd for C_28_H_47_O_16_, 639.2870) in the HRESI-TOF-MS spectrum. In addition to signals due to α-d-glucopyranosyl(1→4)-β-d-glucopyranosyl (δ 5.05 (1H, d, *J* = 8.0 Hz, H-1′), 5.84 (1H, d, *J* = 3.5 Hz, H-1″)), like those of **2**, the ^1^H and ^13^C-NMR (Table 4, C_5_D_5_N) spectra (Supplementary data) of **4** exhibited signals assignable to three methyl as well as four oxygenated carbons, including one methylene, two methine, and one quaternary carbon. However, no signal due to an olefinic bond presented in its ^13^C-NMR spectrum. Combined with the HRESI-TOF-MS experiment, the unsaturation degree for aglycon of **4** was three. Hence, three ring systems were deduced to exist in its aglycon. Comparing its ^13^C-NMR spectrum with that of **2**, the chemical shift of C-14 shifted to a significantly lower field (δ_C_ 64.4 for **2**; 70.4 for **4**, in C_5_D_5_N). Moreover, the long-range correlation from δ_H_ 3.93, 4.42 (H_2_-14) to δ_C_ 83.4 (C-4) was found in the HMBC spectrum. According to the abovementioned two pieces of evidence, an ether bond had to be located at C-4 and C-14 in **4**. Furthermore, its planar structure was identified by the proton and proton correlations and the long-range correlations from proton to carbon exhibited in the ^1^H–^1^H COSY and HMBC spectrum (Figure 2), respectively. The ^1^H and ^13^C-NMR spectra of **4** were in accordance with those of dictamnoside A (**10**), whose configuration was determined by X-ray diffraction analysis [7]. Meanwhile, C-4′ was substituted by an α-d-glucopyranosyl group in **4**. Furthermore, NOE correlations were observed between the following proton and proton pairs: δ_H_ 3.80 (H-1) and δ_H_ 2.02 (Hβ-10), 2.59 (H-5); δ_H_ 2.59 (H-5) and δ_H_ 1.42 (H_3_-12), 1.66 (H_3_-15); δ_H_ 2.04 (H-7) and δ_H_ 4.42 (H-14b); δ_H_ 2.02 (Hβ-10) and δ_H_ 1.42 (H_3_-12), 1.60 (H_3_-13), as shown in Figure 3. Finally, its structure was identified as 4α,10α-epoxy-5β,7α(*H*)-eudesmane-1α,6β,11-triol 6-*O*-α-d-glucopyranosyl(1→4)-β-d-glucopyranoside.

Dictameudesmnoside D (**5**) was obtained as a white powder with positive optical rotation ([α${]}_{D}^{25}$+ 79.0). It was determined to possess the molecular formula C_27_H_44_O_13_ by its quasi-molecular ion peak at *m/z* 621.2739 [M + COOH]^−^ (calcd for C_28_H_45_O_15_, 621.2764) in the negative HRESI-TOF-MS experiment, which was 16 amu smaller than that of **4**. Meanwhile, comparing the ^1^H and ^13^C-NMR (Table 5, CD_3_OD) spectra (Supplementary data) of **5** with those of **4** suggested that **5** had one tri-substituted olefinic group (δ 5.78 (1H, d, *J* = 1.0 Hz, H-6)) more and one methylene as well as one oxygenated methine less than **4**. The olefinic bond should be located at C-5/C-6 in **4** from a biogenetic point of view, which was clarified by the long-range correlations from δ_H_ 1.31 (H_3_-15) to δ_C_ 40.2 (C-3), 81.4 (C-4), 147.1 (C-5); δ_H_ 1.38, 1.77 (H_2_-8), 3.24, 4.20 (H_2_-14), 5.78 (H-6) to δ_C_ 49.4 (C-10) observed in the HMBC experiment. Moreover, comparing the ^1^H and ^13^C-NMR data with those of dictamnoside L (**11**), its aglycon was determined to be 4α,10α-epoxy-5β,7α(*H*)-eudesm-5-ene-1α,6β,11-triol. Its relative configuration was clarified by the NOE correlations shown in the NOESY experiment (Figure 3). On the other hand, the ^1^H and ^13^C-NMR signals due to the sugar moiety of **5** were in good agreement with those of **3**, which indicated that α-d-glucopyranosyl(1→6)-β-d-glucopyranosyl (δ 4.58 (1H, d, *J* = 8.0 Hz, H-1′), 4.83 (1H, d, *J* = 3.0 Hz, H-1″)) should be present in **5**, too. Furthermore, the long-range correlation from δ_H_ 4.58 (H-1′) to δ_C_ 81.7 (C-11) exhibited in the HMBC spectrum suggested that α-d-glucopyranosyl(1→6)-β-d-glucopyranosyl was attached to the C-11 position of the aglycon. On the basis of the abovementioned evidence, the structure of dictameudesmnoside D (**5**) was formulated as 4α,10α-epoxy-5β,7α(*H*)-eudesm-5-ene-1α,6β,11-triol 11-*O*-α-d-glucopyranosyl(1→6)-β-d-glucopyranoside.

The molecular formula of dictameudesmnoside E (**6**) was assigned as C_27_H_46_O_13_ on the basis of the ^13^C-NMR data and negative-ion HRESI-TOF-MS (*m/z* 623.2937 [M + COOH]^−^; calcd for C_28_H_47_O_15_, 623.2920) experiment, which was 16 amu less than that of **3**, suggesting that the difference between them was one oxygen atom on the elementary composition. The treatment of **6** with 1 M HCl liberated d-glucose, which was identified by HPLC analysis using an optical rotation detector [12]. The ^1^H and ^13^C-NMR (Table 6, CD_3_OD) spectra (Supplementary data) and various 2D-NMR (^1^H–^1^H COSY, HSQC, HMBC) spectra indicated the presence of two β-d-glucopyranosyl groups (δ 4.31 (1H, d, *J* = 7.5 Hz, H-1′), 4.54 (1H, d, *J* = 8.0 Hz, H-1″)). Meanwhile, its aglycon was similar to that of **3**. However, **6** had one methyl group more and one hydroxymethyl less than **3**. In the HMBC experiment, long-range correlations were observed from the following proton and carbon pairs: δ_H_ 3.65 (H-1) to δ_C_ 53.8 (C-5); δ_H_ 2.05, 2.51 (H_2_-2) to δ_C_ 39.2 (C-10); δ_H_ 5.29 (H-3) to δ_C_ 53.8 (C-5); δ_H_ 4.55 (H-6) to δ_C_ 39.2 (C-10), 135.8 (C-4); δ_H_ 1.75, 1.99 (H_2_-8) to δ_C_ 39.2 (C-10); δ_H_ 1.24 (H_3_-12) to δ_C_ 30.2 (C-13), 45.0 (C-7), 73.9 (C-11); δ_H_ 1.33 (H_3_-13) to δ_C_ 29.5 (C-12), 45.0 (C-7), 73.9 (C-11); δ_H_ 0.93 (H_3_-14) to δ_C_ 30.6 (C-9), 39.2 (C-10), 53.8 (C-5), 87.5 (C-1); δ_H_ 1.82 (H_3_-15) to δ_C_ 53.8 (C-5), 122.7 (C-3), 135.8 (C-4); δ_H_ 4.31 (H-1′) to δ_C_ 87.5 (C-1); δ_H_ 4.54 (H-1″) to δ_C_ 80.4 (C-6). Then, the planar structure of dictameudesmnoside E (**6**) was deduced. Moreover, its relative configuration was elucidated by the NOE correlations between H-5 and H-1, H_3_-12; H-7 and H-6, H_3_-14 observed in its NOESY experiment. Consequently, the structure of **6** was identified as 5β,7α(*H*),10α-eudesm-3-ene-1α,6β,11-triol 1-*O*-β-d-glucopyranosyl-6-*O*-β-d-glucopyranoside.

The structures of known compounds **7**–**13** were identified by comparing their ^1^H and ^13^C-NMR data with those of references.

**Figure 4:**
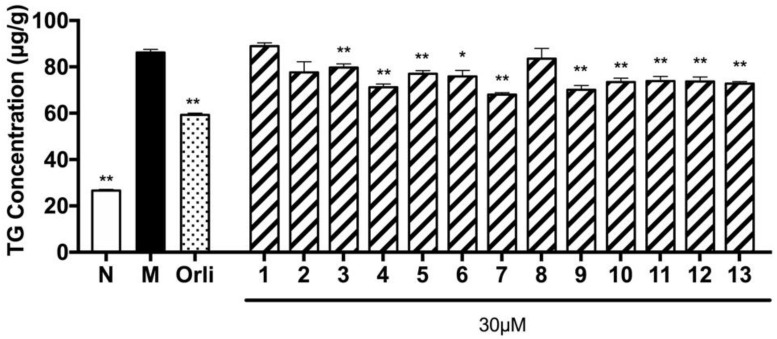
Effects of compounds **1**–**13** on TG overloading in HepG2 cells. Cells were treated with 200 µmol/L SO for 48 h. Meanwhile, 30 µmol/L tested compounds or 5 µmol/L orlistat (Orli.) were co-incubated to evaluate their inhibitory effects, respectively. Each value represents the mean ± S.E.M., *n* = 5, ** *p* < 0.01, * *p* < 0.05 vs. model group (Mod.). Nor. = normal group.

In addition, TG accumulation inhibitory effects were screened by the sodium oleate (SO) induced hepatic cell line method [13]. As shown in Figure 4, compounds **3**–**5**, **7**, and **9**–**13** obtained from the 70% EtOH extract of *D. dasycarpus* root bark significantly inhibited TG accumulation in HepG2 cells.

## 3. Experimental

### 3.1. General

Optical rotations were measured on a Rudolph Autopol^®^ IV automatic polarimeter (*l* = 50 mm) (Rudolph Research Analytical, Hackettstown, NJ, USA). IR spectra were recorded on a Varian 640-IR FT-IR spectrophotometer (Varian Australia Pty Ltd., Mulgrave, Australia). NMR spectra were determined on a Bruker 500 MHz NMR spectrometer (Bruker BioSpin AG Industriestrasse 26 CH-8117, Fällanden, Switzerland) at 500 MHz for ^1^H and 125 MHz for ^13^C-NMR (internal standard: TMS). Negative-ion mode HRESI-TOF-MS results were obtained on an Agilent Technologies 6520 Accurate-Mass Q-Tof LC/MS spectrometer (Agilent Corp., Santa Clara, CA, USA).

CC was performed on macroporous resin D101 (Haiguang Chemical Co., Ltd., Tianjin, China), silica gel (48–75 μm, Qingdao Haiyang Chemical Co., Ltd., Qingdao, China), and ODS (40–63 μm, YMC Co., Ltd., Tokyo, Japan). pHPLC columns, Cosmosil 5C_18_-MS-II (20 mm i.d. × 250 mm, Nacalai Tesque, Inc., Kyoto, Japan), and Cosmosil PBr (20 mm i.d. × 250 mm, Nacalai Tesque, Inc., Kyoto, Japan) were used to separate the constituents.

### 3.2. Plant Material

The root barks of *Dictamnus* *dasycarpus* Turcz. were purchased from the medicine market in Anguo city, Heibei province, China, and identified by Dr. Li Tianxiang (Experiment Teaching Department, Tianjin University of Traditional Chinese Medicine). The voucher specimen has been deposited at the Academy of Traditional Chinese Medicine of Tianjin University of TCM.

### 3.3. Extraction and Isolation

The root barks of *D. dasycarpus* (9.0 kg) were refluxed with 70% ethanol-water. Then, the 70% EtOH extract (1725.3 g) was partitioned in an EtOAc–H_2_O mixture (1:1, *v*/*v*). The H_2_O layer (1253.8 g) was subjected to D101 macroporous resin CC (H_2_O → 95% EtOH). As a result, H_2_O (1032.9 g) and 95% EtOH (123.4 g) eluates were obtained. The 95% EtOH eluate (90.0 g) was subjected to silica gel CC (CHCl_3_ → CHCl_3_–MeOH (100:1 → 100:3 → 100:7, *v*/*v*) → CHCl_3_–MeOH–H_2_O (10:3:1 → 7:3:1 → 6:4:1, *v*/*v*/*v*, lower layer) → MeOH), and 12 fractions (Fr. 1–Fr. 12) were given. Fraction 8 (12.1 g) was isolated by ODS CC (MeOH–H_2_O (10% → 20% → 30% → 40% → 50% → 60% → 100%)) to yield seven fractions (Fr. 8-1–Fr. 8-7). Fraction 8-2 (4.4 g) was separated by pHPLC (CH_3_CN–H_2_O (8:92, *v*/*v*) + 1% HAc, Cosmosil 5C18-MS-II column) to produce 11 fractions (Fr. 8-2-1–Fr. 8-2-11). Fraction 8-2-6 (575.1 mg) was further purified by pHPLC (CH_3_CN–H_2_O (17:83, *v*/*v*), Cosmosil PBr column) to gain dictamnoside A (**10**, 69.4 mg). Fraction 8-2-9 (379.3 mg) was isolated by pHPLC (CH_3_CN–H_2_O (17:83, *v*/*v*), Cosmosil PBr column) to yield dictamnoside B (**7**, 18.2 mg). Fraction 8-2-11 (923.2 mg) was separated by pHPLC (CH_3_CN–H_2_O (17:83, *v*/*v*), Cosmosil PBr column), and dictamnoside L (**11**, 122.8 mg) was given. Fraction 8-4 (1.01 g) was purified by pHPLC (CH_3_CN–H_2_O (20:80, *v*/*v*) + 1% HAc, Cosmosil PBr column) to provide 18 fractions (Fr. 8-4-1–Fr. 8-4-18). Fraction 8-4-1 (233.2 mg) was further isolated by pHPLC (CH_3_CN–H_2_O (16:84, *v*/*v*) + 1% HAc, Cosmosil PBr column) to yield dictamnoside C (**9**, 160.2 mg). Fraction 10 (10.1 g) was subjected to ODS CC (MeOH–H_2_O (10% → 20% → 30% → 40% → 50% → 60% → 80% → 100%, *v*/*v*)) to provide eight fractions (Fr. 10-1–Fr. 10-8). Fraction 10-2 (1.2 g) was separated by pHPLC (MeOH–H_2_O (20:80, *v*/*v*) + 1% HAc, Cosmosil 5C18-MS-II column), and then by pHPLC (CH_3_CN–H_2_O (8:92, *v*/*v*), Cosmosil 5C18-MS-II column) to produce dictamnoside D (**12**, 12.8 mg). Fraction 10-3 (2.6 g) was isolated by pHPLC (CH_3_CN–H_2_O (9:91, *v*/*v*), Cosmosil 5C18-MS-II column), and 16 fractions (Fr. 10-3-1–Fr. 10-3-16) were given. Fraction 10-3-4 was identified as dictamnoside K (**13**, 88.0 mg). Fraction 10-3-6 (71.7 mg) was purified by pHPLC (CH_3_CN–H_2_O (11:89, *v*/*v*), Cosmosil PBr column) to obtain dictameudesmnoside C (**4**, 17.4 mg). Using the same isolation condition, five fractions (Fr. 10-3-11-1–Fr. 10-3-11-5) were produced from fraction 10-3-11 (305.3 mg). Among them, fractions 10-3-11-1 and 10-3-11-5 were elucidated to be dictamnoside G (**8**, 179.6 mg) and dictameudesmnoside B (**3**, 26.7 mg), respectively. Fraction 10-3-11-2 (20.6 mg) was subjected to silica gel CC (CHCl_3_–MeOH–H_2_O (20:3:1 → 15:3:1 → 10:3:1, *v*/*v*/*v*, lower layer) → MeOH) to yield dictameudesmnoside A_2_ (**2**, 20.6 mg). Fraction 10-3-13 (130.5 mg) was separated by pHPLC (CH_3_CN–H_2_O (15:85, *v*/*v*), Cosmosil PBr column), and finally by silica gel (CHCl_3_–MeOH–H_2_O (15:3:1 → 10:3:1, *v*/*v*/*v*, lower layer) → MeOH) to afford dictameudesmnoside D (**5**, 38.7 mg). Fraction 10-3-14 (191.0 mg) was isolated by pHPLC (CH_3_CN–H_2_O (15:85, *v*/*v*), Cosmosil PBr column) to yield seven fractions (Fr. 10-3-14-1–Fr. 10-3-14-7). Fraction 10-3-14-4 (30.0 mg) was further purified by silica gel CC (CHCl_3_–MeOH–H_2_O (15:3:1 → 10:3:1, *v*/*v*/*v*, lower layer) → MeOH) to give dictameudesmnoside A_1_ (**1**, 24.9 mg). Fraction 10-7 (2.2 g) was subjected to Sephadex LH-20 CC (MeOH), and two fractions (Fr. 10-7-1–Fr. 10-7-2) were given. Fraction 10-7-2 (1.41 g) was purified by pHPLC (MeOH–H_2_O (45:55, *v*/*v*), Cosmosil 5C18-MS-II column) to afford dictameudesmnoside E (**6**, 6.0 mg).

Dictameudesmnoside *A*_1_ *(***1***):* White powder; [α${]}_{D}^{25}$ + 53.5 (*c* = 0.77, MeOH); IR *ν*_max_ (KBr) cm^−1^: 3372, 2931, 1650, 1601, 1455, 1380, 1150, 1075, 1037; ^1^H-NMR (500 MHz, CD_3_OD/C_5_D_5_N) and ^13^C-NMR (125 MHz, CD_3_OD/C_5_D_5_N) spectroscopy data: see Table 1. HRESI-TOF-MS: Negative-ion mode *m/z* 593.2826 [M − H]^−^ (calcd for C_27_H_45_O_14_, 593.2815).

Dictameudesmnoside *A_2_ (***2***):* White powder; [α${]}_{D}^{25}$ + 32.1 (*c* = 0.86, MeOH); IR *ν*_max_ (KBr) cm^−1^: 3381, 2931, 1647, 1602, 1452, 1381, 1147, 1071, 1033; ^1^H-NMR (500 MHz, CD_3_OD/C_5_D_5_N) and ^13^C-NMR (125 MHz, CD_3_OD/C_5_D_5_N) spectroscopy data: see Table 2. HRESI-TOF-MS: Negative-ion mode *m/z* 639.2883 [M + COOH]^−^ (calcd for C_28_H_47_O_16_, 639.2870).

Dictameudesmnoside *B (***3***):* White powder; [α${]}_{D}^{25}$ + 23.6 (*c* = 0.66, MeOH); IR *ν*_max_ (KBr) cm^−1^: 3394, 2925, 1636, 1604, 1451, 1371, 1152, 1075, 1035; ^1^H-NMR (500 MHz, CD_3_OD) and ^13^C-NMR (125 MHz, CD_3_OD) spectroscopy data: see Table 3. HRESI-TOF-MS: Negative-ion mode *m/z* 639.2886 [M + COOH]^−^ (calcd for C_28_H_47_O_16_, 639.2870).

Dictameudesmnoside *C (***4***):* White powder; [α${]}_{D}^{25}$ + 27.3 (*c* = 0.63, MeOH); IR *ν*_max_ (KBr) cm^−1^: 3331, 2934, 1455, 1383, 1150, 1073, 1027. ^1^H-NMR (500 MHz, C_5_D_5_N) and ^13^C-NMR (125 MHz, C_5_D_5_N) spectroscopy data: see Table 4. HRESI-TOF-MS: Negative-ion mode *m/z* 639.2878 [M + COOH]^−^ (calcd for C_28_H_47_O_16_, 639.2870).

Dictameudesmnoside *D (***5***):* White powder; [α${]}_{D}^{25}$ + 79.0 (*c* = 0.82, MeOH); IR *ν*_max_ (KBr) cm^−1^: 3385, 2934, 2878, 1635, 1601, 1453, 1379, 1152, 1079, 1028; ^1^H-NMR (500 MHz, CD_3_OD) and ^13^C-NMR (125 MHz, CD_3_OD) spectroscopy data: see Table 5. HRESI-TOF-MS: Negative-ion mode *m/z* 621.2739 [M + COOH]^−^ (calcd for C_28_H_45_O_15_, 621.2764).

Dictameudesmnoside *E (***6***):* White powder; [α${]}_{D}^{25}$ − 15.3 (*c* = 0.30, MeOH); IR *ν*_max_ (KBr) cm^−1^: 3370, 2927, 1627, 1449, 1388, 1155, 1075, 1042, 1018; ^1^H-NMR (500 MHz, CD_3_OD/D_2_O) and ^13^C-NMR (125 MHz, CD_3_OD/D_2_O) spectroscopy data: see Table 6; HRESI-TOF-MS: Negative-ion mode *m/z* 623.2937 [M + COOH]^−^ (calcd for C_28_H_47_O_15_, 623.2920).

### 3.4. Acid Hydrolysis of **1**–**6**

A solution of each of the new eudesmane-type sesquiterpene glycosides **1**–**6** (about 3.0 mg) in 1 M HCl (1 mL) was heated under reflux for 3 h, respectively. Then each reaction mixture was neutralized with Amberlite IRA-400 (OH^−^ form) and removed by filtration. The aqueous layer was subjected to HPLC analysis: HPLC column, Kaseisorb LC NH_2_-60-5, 4.6 mm i.d. × 250 mm (Tokyo Kasei Co., Ltd., Tokyo, Japan); detection, optical rotation (Shodex OR-2 (Showa Denko Co., Ltd., Tokyo, Japan); mobile phase, CH_3_CN–H_2_O ((75:25, *v*/*v*; flow rate 1.0 mL/min)). From the results, instances d-glucose (12.8 min, positive optical rotation) for **1**–**6** were identified by comparison of their retention times and optical rotations with that of an authentic sample.

### 3.5. Inhibitory Effects of Sodium Oleate-Induced Lipid Accumulation in HepG2 Cells

Materials: HepG2 cell line was obtained from the Cell Resource Center of the Institute of Basic Medical Sciences, Chinese Academy of Medical Sciences and Peking Union Medical College (Beijing, China). Fetal Bovine Serum (FBS) was purchased from Biological Industries (Beit-Haemek, Israel). Dulbecco’s modified Eagle’s medium (DMEM), penicillin G sodium salt, and streptomycin sulfate were ordered from Thermo SCIENTIFIC (Waltham, MA, USA). TG assay kit was purchased from BioSino Bio-technology and Science Inc. (Beijing, China). SO and orlistat were obtained from Sigma-Aldrich Corporation (St. Louis, MO, USA).

Cell culture: HepG2 cells were routinely cultured in DMEM-based medium as described before [13]. After cells reached about 80% confluence, seeded at a density of 80,000 cells/mL in 48-well plates for 24 h, the experiments were performed.

Sodium oleate-induced lipid accumulation: To induce the overloading of intracellular lipid, SO was used as previous reported. Briefly, after being seeded in 48-well plates in FBS-free medium for 24 h, HepG2 cells were exposed to 200 µM SO in the presence or absence of isolates (30 µM) or the positive control, orlistat (5 µM), for another 48 h. The intracellular TG content was finally examined using a commercial assay kit at 492 nm after cells were rinsed with phosphate-buffered saline (PBS).

Statistical analysis: Values are expressed as means ± S.D. All of the grouped data were statistically analyzed with SPSS 11.0. Significant differences between means were evaluated by one-way analysis of variance (ANOVA), and Tukey’s Studentized range test was used for post hoc evaluations. *p* < 0.05 was considered to indicate statistical significance.

## 4. Conclusions

In conclusion, 13 eudesmane-type sesquiterpene glycosides have been isolated from the 70% EtOH extract of *D. dasycarpus* root bark. Their structures have been determined on the basis of spectroscopic and chemical analyses. The existence of the α-configuration glucose units in their structures (e.g., **1**–**5**, **8**) is not very common in natural glycosidic components.

On the other hand, as one of main constituents of *D. dasycarpus* root bark, the eudesmane-type sesquiterpene glycosides, isolates **3**–**5**, **7**, and **9**–**13**, showed significant TG accumulation inhibitory activity, which was identical to the information reported by Kim et al. that the decoction could reduce TG and total cholesterol contents in blood as well as alleviate obesity effectively [10]. In addition, we can deduce that *D. dasycarpus* is one of potential active plants for improving metabolic disease.

## Figures and Tables

**Table 1:** ^1^H and ^13^C-NMR data for **1** in CD_3_OD and C_5_D_5_N.

No.	In CD_3_OD		In C_5_D_5_N	
	δ_C_	δ_H_ (*J* in Hz)	δ_C_	δ_H_ (*J* in Hz)
1	82.4	3.60 (m, overlapped)	81.5	3.65 (dd, 5.0, 11.0)
2	33.9	1.75, 1.94 (both m)	33.9	2.08 (m)
3	36.8	2.22, 2.33 (both m)	36.3	2.35 (m)
4	147.7	―	147.3	―
5	51.8	2.55 (d, 6.5)	51.2	2.78 (d, 7.0)
6	78.4	4.51 (dd, 5.0, 6.5)	77.5	4.91 (dd, 5.5, 6.5)
7	46.7	1.98 (m)	46.2	2.32 (m)
8	20.3	1.62, 1.83 (both m)	20.3	1.76, 2.02 (both m)
9	28.7	1.59, 2.25 (both m)	28.9	1.89, 2.78 (both m)
10	43.5	―	43.1	―
11	74.3	―	72.7	―
12	30.6	1.31 (s)	31.3	1.49 (s)
13	28.7	1.33 (s)	28.5	1.53 (s)
14	64.4	3.45, 3.88 (both d, 11.5)	63.9	3.82, 4.31 (both d, 11.0)
15	107.5	4.88, 4.92 (both br. s)	106.7	4.97, 5.08 (both br. s)
1′	103.9	4.47 (d, 8.0)	103.8	4.98 (d, 7.5)
2′	74.0	3.26 (dd, 9.0, 9.0)	73.5	3.88 (dd, 7.5, 9.0)
3′	88.0	3.43 (dd, 9.0, 9.0)	88.2	4.11 (dd, 9.0, 9.0)
4′	70.5	3.60 (m, overlapped)	70.4	4.36 (dd, 9.0, 9.0)
5′	77.3	3.06 (m)	77.5	3.57 (m)
6′	62.0	3.70 (m)	61.8	4.27 (m)
1″	101.5	5.12 (d, 3.5)	102.1	5.84 (d, 4.0)
2″	74.0	3.44 (dd, 3.5, 9.0)	74.3	4.17 (dd, 4.0, 9.0)
3″	75.1	3.65 (dd, 9.0, 9.0)	75.6	4.57 (dd, 9.0, 9.0)
4″	71.8	3.27 (dd, 9.0, 9.0)	72.0	4.18 (dd, 9.0, 9.0)
5″	74.2	3.87 (m)	74.5	4.76 (m)
6″	62.6	3.63 (dd, 4.0, 12.0)	62.5	4.29 (dd, 6.0, 12.0)
		3.82 (dd, 1.5, 12.0)		4.47 (dd, 2.5, 12.0)

**Table 2:** ^1^H and ^13^C-NMR data for **2** in CD_3_OD and C_5_D_5_N.

No.	In CD_3_OD		In C_5_D_5_N	
	δ_C_	δ_H_ (*J* in Hz)	δ_C_	δ_H_ (*J* in Hz)
1	82.4	3.62 (m)	81.6	3.62 (m)
2	33.9	1.74, 1.94 (both m)	34.0	2.05 (m)
3	36.8	2.23, 2.34 (both m)	36.3	2.35 (m)
4	147.7	―	147.7	―
5	51.9	2.55 (d, 6.5)	51.5	2.75 (d, 7.0)
6	78.4	4.50 (dd, 5.0, 6.5)	78.0	4.92 (m, overlapped)
7	46.7	1.98 (m)	46.6	2.29 (m)
8	20.3	1.62, 1.83 (both m)	20.2	1.77, 2.11 (both m)
9	28.8	1.61, 2.25 (both m)	28.9	1.96, 2.79 (both m)
10	43.5	―	42.9	―
11	74.3	―	72.6	―
12	30.6	1.31 (s)	31.3	1.54 (s)
13	28.6	1.32 (s)	28.7	1.59 (s)
14	64.5	3.44, 3.87 (both d, 12.0)	64.4	3.79, 4.32 (both d, 11.0)
15	107.5	4.88, 4.91 (both br. s)	106.3	4.96, 5.01 (both br. s)
1′	104.1	4.44 (d, 8.0)	104.5	4.90 (d, 8.0)
2′	74.8	3.17 (dd, 8.0, 9.0)	74.7	3.91 (dd, 8.0, 8.5)
3′	78.3	3.55 (dd, 9.0, 9.0)	78.3	4.24 (m, overlapped)
4′	80.8	3.56 (dd, 9.0, 9.0)	80.7	4.41 (dd, 9.5, 9.5)
5′	76.5	3.14 (m)	76.5	3.49 (m)
6′	61.7	3.73 (dd, 2.0, 12.0)	61.4	4.23 (m, overlapped)
		3.81 (dd, 4.5, 12.0)		4.44 (dd, 2.5, 12.0)
1″	102.8	5.14 (d, 3.5)	103.0	5.91 (d, 4.0)
2″	74.2	3.43 (dd, 3.5, 10.0)	74.5	4.16 (dd, 4.0, 9.0)
3″	75.1	3.58 (dd, 9.0, 9.0)	75.5	4.59 (dd, 9.0, 9.0)
4″	71.6	3.24 (dd, 9.5, 10.0)	72.0	4.14 (dd, 9.0, 9.0)
5″	74.8	3.67 (m)	75.3	4.56 (m, overlapped)
6″	62.9	3.64 (dd, 4.0, 12.0)	62.8	4.33 (dd, 7.0, 12.0)
		3.82 (br. d, ca. 12)		4.56 (m, overlapped)

**Table 3:** ^1^H and ^13^C-NMR data for **3** in CD_3_OD.

No.	δ_C_	δ_H_ (*J* in Hz)	No.	δ_C_	δ_H_ (*J* in Hz)
1	79.6	3.76 (m)			3.96 (d, 11.0)
2	35.0	2.12, 2.52 (both m)	15	23.0	1.83 (s)
3	122.3	5.31 (br. s)	1′	103.9	4.60 (d, 8.0)
4	135.2	―	2′	75.2	3.20 (dd, 8.0, 8.5)
5	51.0	2.92 (br. s)	3′	78.3	3.36 (m, overlapped)
6	79.4	4.49 (dd, 3.5, 4.5)	4′	71.6	3.33 (dd, 9.0, 9.0)
7	45.3	1.97 (m)	5′	76.5	3.36 (m, overlapped)
8	18.8	1.83 (m, overlapped)	6′	68.2	3.62 (m, overlapped)
		2.00 (m)			3.95 (dd, 4.5, 12.0)
9	26.6	1.29 (m, overlapped)	1″	100.8	4.80 (d, 4.0)
		2.34 (ddd, 1.5, 8.0, 13.5)	2″	73.8	3.37 (m, overlapped)
10	42.7	―	3″	75.3	3.64 (dd, 9.5, 9.5)
11	74.2	―	4″	71.5	3.37 (m, overlapped)
12	29.8	1.30 (s)	5″	73.6	3.61 (m)
13	30.4	1.33 (s)	6″	62.5	3.70 (m, overlapped)
14	62.5	3.70 (m, overlapped)			3.78 (dd, 2.5, 12.0)

**Table 4:** ^1^H and ^13^C-NMR data for **4** in C_5_D_5_N.

No.	δ_C_	δ_H_ (*J* in Hz)	No.	δ_C_	δ_H_ (*J* in Hz)
1	76.1	3.80 (m)	15	22.7	1.66 (s)
2	30.4	2.06 (m)	1′	106.7	5.05 (d, 8.0)
3	41.1	1.81, 1.92 (both m)	2′	74.5	3.97 (dd, 8.0, 8.0)
4	83.4	―	3′	78.2	4.30 (dd, 8.0, 8.0)
5	56.4	2.59 (br. s)	4′	81.5	4.24 (dd, 9.0, 9.0)
6	79.3	4.83 (br. s)	5′	77.0	3.78 (m)
7	44.2	2.04 (m)	6′	62.1	4.44 (m)
8	17.7	1.84, 2.43 (both m)	1″	103.0	5.84 (d, 3.5)
9	23.0	2.02, 2.25 (both m)	2″	74.4	4.16 (dd, 3.5, 9.5)
10	50.0	―	3″	75.5	4.58 (dd, 9.5, 9.5)
11	72.1	―	4″	71.9	4.14 (dd, 9.5, 9.5)
12	29.5	1.42 (s)	5″	75.3	4.55 (m)
13	30.6	1.60 (s)	6″	62.8	4.31 (dd, 6.0, 12.0)
14	70.4	3.93, 4.42 (both d, 8.5)			4.54 (dd, 1.5, 12.0)

**Table 5:** ^1^H and ^13^C-NMR data for **5** in CD_3_OD.

No.	δ_C_	δ_H_ (*J* in Hz)	No.	δ_C_	δ_H_ (*J* in Hz)
1	72	3.78 (m)	15	21.3	1.31 (s)
2	29.9	1.72, 1.91 (both m)	1′	98.7	4.58 (d, 8.0)
3	40.2	1.44, 1.70 (both m)	2′	75.3	3.20 (dd, 8.0, 8.0)
4	81.4	―	3′	78.4	3.39 (dd, 8.0, 9.0)
5	147.1	―	4′	71.5	3.43 (dd, 9.0, 9.0)
6	116.5	5.78 (d, 1.0)	5′	76.0	3.48 (m)
7	46.7	2.48 (ddd, 1.0, 5.0, 12.0)	6′	67.5	3.62 (dd, 3.0, 12.0)
8	21.7	1.38, 1.77 (both m)			3.95 (dd, 4.5, 12.0)
9	26.3	1.22 (ddd, 3.0, 13.0, 13.0)	1″	100.0	4.83 (d, 3.0)
		2.19 (ddd, 2.0, 2.0, 13.0)	2″	73.8	3.36 (dd, 3.0, 9.5)
10	49.4	―	3″	75.3	3.64 (m, overlapped)
11	81.7	―	4″	71.7	3.33 (dd, 9.5, 9.5)
12	25.6	1.30 (s)	5″	73.5	3.65 (m, overlapped)
13	22.6	1.17 (s)	6″	62.6	3.68 (dd, 5.5, 12.0)
14	74.8	3.24, 4.20 (both d, 7.5)			3.80 (dd, 2.0, 12.0)

**Table 6:** ^1^H and ^13^C-NMR data for **6** in CD_3_OD and D_2_O.

No.	In CD_3_OD		In D_2_O	
	δ_C_	δ_H_ (*J* in Hz)	δ_C_	δ_H_ (*J* in Hz)
1	87.5	3.65 (m, overlapped)	90.2	3.77 (dd, 7.5, 11.5)
2	34.0	2.05, 2.51 (both m)	35.1	2.07, 2.50 (both m)
3	122.7	5.29 (br. s)	124.0	5.40 (br. s)
4	135.8	―	137.7	―
5	53.8	2.89 (br. s)	56.0	2.78 (br. s)
6	80.4	4.55 (t, 4.0)	83.4	4.66 (t, 2.0)
7	45.0	1.85 (m)	45.6	1.90 (m)
8	17.9	1.75, 1.99 (both m)	18.2	1.85 (m)
9	30.6	1.30, 2.18 (both m)	31.9	1.33, 2.13 (both m)
10	39.2	―	40.0	―
11	73.9	―	76.6	―
12	29.5	1.24 (s)	30.3	1.24 (s)
13	30.2	1.33 (s)	31.2	1.38 (s)
14	13.0	0.93 (s)	15.0	0.91 (s)
15	22.9	1.82 (s)	24.0	1.82 (s)
1′	106.0	4.31 (d, 7.5)	106.7	4.51 (d, 8.0)
2′	75.7	3.15 (dd, 7.5, 9.0)	76.3	3.27 (dd, 8.0, 8.5)
3′	78.2	3.35 (m, overlapped)	78.9	3.50 (dd, 8.5, 9.0)
4′	71.8	3.26 (dd, 9.0, 9.0)	72.4	3.43 (dd, 9.0, 9.0)
5′	77.8	3.24 (m, overlapped)	78.6	3.43 (m, overlapped)
6′	62.9	3.65 (m, overlapped)	63.4	3.72 (dd, 4.5, 12.0)
		3.85 (dd, 1.5, 11.5)		3.82 (dd, 2.0, 12.0)
1″	105.4	4.54 (d, 8.0)	106.7	4.73 (d, 8.0)
2″	75.2	3.18 (dd, 8.0, 9.0)	76.5	3.27 (dd, 8.0, 8.5)
3″	78.5	3.35 (m, overlapped)	78.7	3.48 (dd, 8.5, 9.0)
4″	71.4	3.35 (m, overlapped)	72.7	3.36 (dd, 9.0, 9.0)
5″	77.8	3.22 (m, overlapped)	78.6	3.43 (m, overlapped)
6″	62.6	3.70 (dd, 4.5, 12.0)	63.7	3.70 (dd, 6.0, 12.0)
		3.76 (dd, 2.0, 12.0)		3.90 (dd, 2.0, 12.0)

## Supplementary Materials

Supplementary materials are available on line.
