# Supplementary material for: Eudesmane-Type Sesquiterpene Glycosides from Dictamnus dasycarpus Turcz
Source: Molecules. 2018 Mar 13;23(3):642. doi: 10.3390/molecules23030642 (PMC6017781; doi:10.3390/molecules23030642)
Supplement: Supplementary file 1 [file molecules-23-00642-s001.pdf]

Supporting Information

Eudesmane-type sesquiterpene glycosides from *Dictamnus dasycarpus* Turcz.

Shengcai Yang [1], Zheng Li [1], Jianli Wang [2], JingYa Ruan [1], Chang Zheng [2], Peijian Huang [2], Lifeng Han [2], Yi Zhang [1,2,*], and Tao Wang [1,2,*]

[1] Tianjin State Key Laboratory of Modern Chinese Medicine, 312 Anshanxi Road, Nankai District,Tianjin 300193, China; 15122473723@163.com (S.Y.); wo15510977612@163.com (Z.L.); Ruanjy19930919@163.com (J.R.)

[2] Tianjin Key Laboratory of TCM Chemistry and Analysis, Institute of Traditional Chinese Medicine, Tianjin University of Traditional Chinese Medicine, 312 Anshanxi Road, Nankai District, Tianjin 300193, China; wjl15802226160@126.com (J.W.); 18702270347@163.com (C.Z.); hpjforever@sina.com (P.H.); hanlifeng_1@sohu.com (L.H.)

* Correspondence: zhwwxzh@263.net (Y.Z.); wangtao@tjutcm.edu.cn (T.W.); Tel./Fax: +86-22-5959-6163 (Y.Z.); +86-22-59596168 (T.W.)

S1. ^{1}H NMR (500 MHz, CD_3OD) spectrum for **1**. 4
S2. Enlarged ^{1}H NMR (500 MHz, CD_3OD) spectrum 1 for **1**. 4
S3. Enlarged ^{1}H NMR (500 MHz, CD_3OD) spectrum 2 for **1**. 5
S4. ^{13}C NMR (125 MHz, CD_3OD) spectrum for **1**. 5
S5. ^{1}H ^{1}H COSY (CD_3OD) spectrum for **1**. 6
S6. HSQC (CD_3OD) spectrum for **1**. 6
S7. HMBC (CD_3OD) spectrum for **1**. 7
S8. NOESY (CD_3OD) spectrum for **1**. 7
S9. ^{1}H NMR (500 MHz, C_5D_5N) spectrum for **1**. 8
S10. ^{13}C NMR (125 MHz, C_5D_5N) spectrum for **1**. 8
S11. HRESI-TOF-MS spectrum for **1**. 9
S12. ^{1}H NMR (500 MHz, CD_3OD) spectrum for **2**. 10
S13. Enlarged ^{1}H NMR (500 MHz, CD_3OD) spectrum 1 for **2**. 10
S14. Enlarged ^{1}H NMR (500 MHz, CD_3OD) spectrum 2 for **2**. 11
S15. ^{13}C NMR (125 MHz, CD_3OD) spectrum for **2**. 11
S16. ^{1}H ^{1}H COSY (CD_3OD) spectrum for **2**. 12
S17. HSQC (CD_3OD) spectrum for **2**. 12
S18. HMBC (CD_3OD) spectrum for **2**. 13
S19. NOESY (CD_3OD) spectrum for **2**. 13
S20. ^{1}H NMR (500 MHz, C_5D_5N) spectrum for **2**. 14
S21. ^{13}C NMR (125 MHz, C_5D_5N) spectrum for **2**. 14
S22. HRESI-TOF-MS spectrum for **2**. 15
S23. ^{1}H NMR (500 MHz, CD_3OD) spectrum for **3**. 16
S24. ^{13}C NMR (125 MHz, CD_3OD) spectrum for **3**. 16
S25. ^{1}H ^{1}H COSY (CD_3OD) spectrum for **3**. 17
S26. HSQC (CD_3OD) spectrum for **3**. 17
S27. HMBC (CD_3OD) spectrum for **3**. 18
S28. NOESY (CD_3OD) spectrum for **3**. 18
S29. HRESI-TOF-MS spectrum for **3**. 19
S30. ^{1}H NMR (500 MHz, C_5D_5N) spectrum for **4**. 20
S31. Enlarged ^{1}H NMR (500 MHz, C_5D_5N) spectrum 1 for **4**. 20
S32. Enlarged ^{1}H NMR (500 MHz, C_5D_5N) spectrum 2 for **4**. 21
S33. ^{13}C NMR (125 MHz, C_5D_5N) spectrum for **4**. 21
S34. ^{1}H ^{1}H COSY (C_5D_5N) spectrum for **4**. 22
S35. HSQC (C_5D_5N) spectrum for **4**. 22
S36. HMBC (C_5D_5N) spectrum for **4**. 23
S37. NOESY (C_5D_5N) spectrum for **4**. 23
S38. HRESI-TOF-MS spectrum for **4**. 24
S39. ^{1}H NMR (500 MHz, CD_3OD) spectrum for **5**. 25
S40. Enlarged ^{1}H NMR (500 MHz, CD_3OD) spectrum 1 for **5**. 25
S41. Enlarged ^{1}H NMR (500 MHz, CD_3OD) spectrum 2 for **5**. 26
S42. ^{13}C NMR (125 MHz, CD_3OD) spectrum for **5**. 26
S43. ^{1}H ^{1}H COSY (CD_3OD) spectrum for **5**. 27
S44. HSQC (CD_3OD) spectrum for **5**. 27

S45. HMBC (CD_3OD) spectrum for **5**. 28
S46. NOESY (CD_3OD) spectrum for **5**. 28
S47. HRESI-TOF-MS spectrum for **5**. 29
S48. ^1H NMR (500 MHz, CD_3OD) spectrum for **6**. 30
S49. Enlarged ^1H NMR (500 MHz, CD_3OD) spectrum 1 for **6**. 30
S50. Enlarged ^1H NMR (500 MHz, CD_3OD) spectrum 2 for **6**. 31
S51. ^{13}C NMR (125 MHz, CD_3OD) spectrum for **6**. 31
S52. ^1H ^1H COSY (CD_3OD) spectrum for **6**. 32
S53. HSQC (CD_3OD) spectrum for **6**. 32
S54. HMBC (CD_3OD) spectrum for **6**. 33
S55. NOESY (CD_3OD) spectrum for **6**. 33
S56. ^1H NMR (500 MHz, D_2O) spectrum for **6**. 34
S57. ^{13}C NMR (125 MHz, D_2O) spectrum for **6**. 34
S58. HRESI-TOF-MS spectrum for **6**. 35

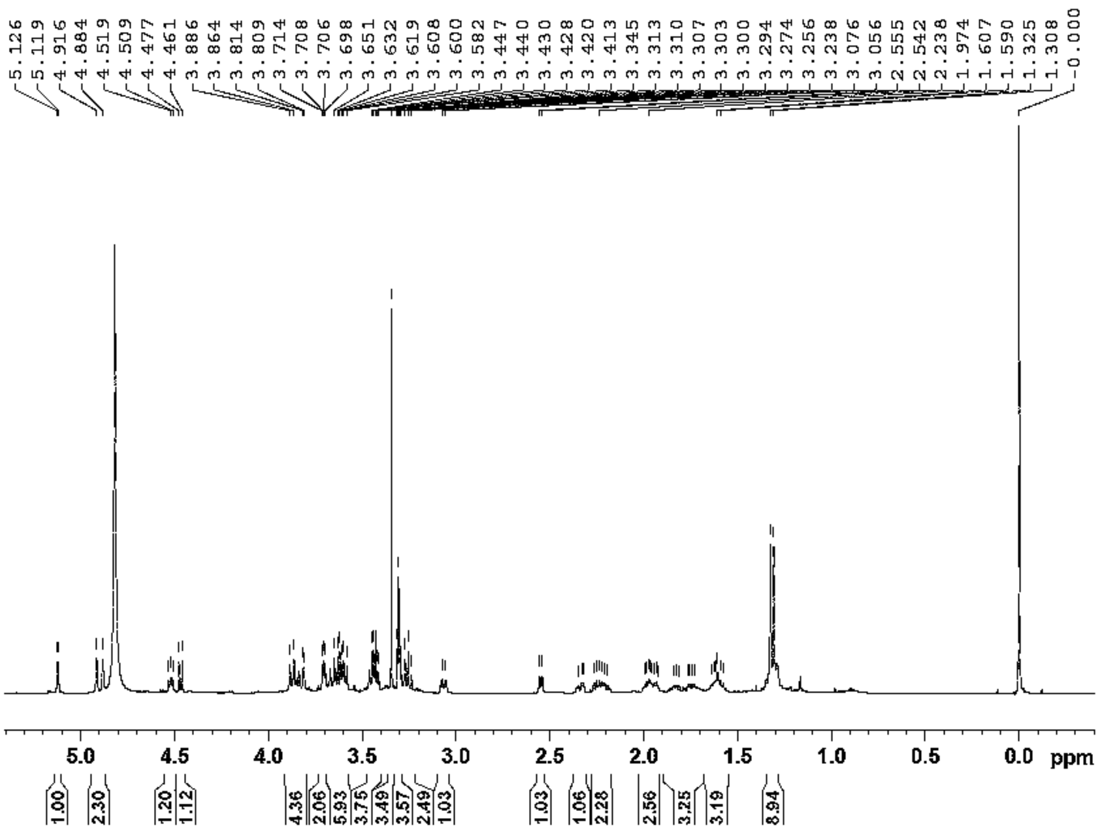

S1. ^{1}H NMR (500 MHz, CD_3OD) spectrum for **1**.

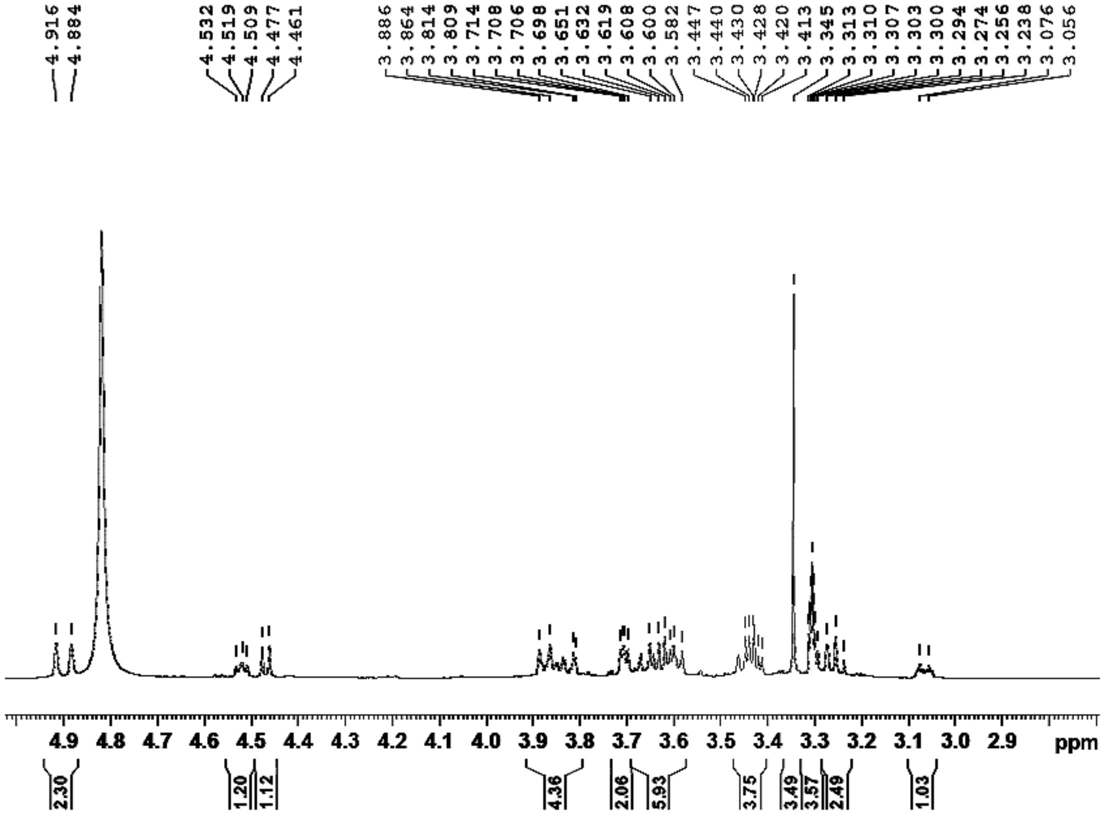

S2. Enlarged ^{1}H NMR (500 MHz, CD_3OD) spectrum 1 for **1**.

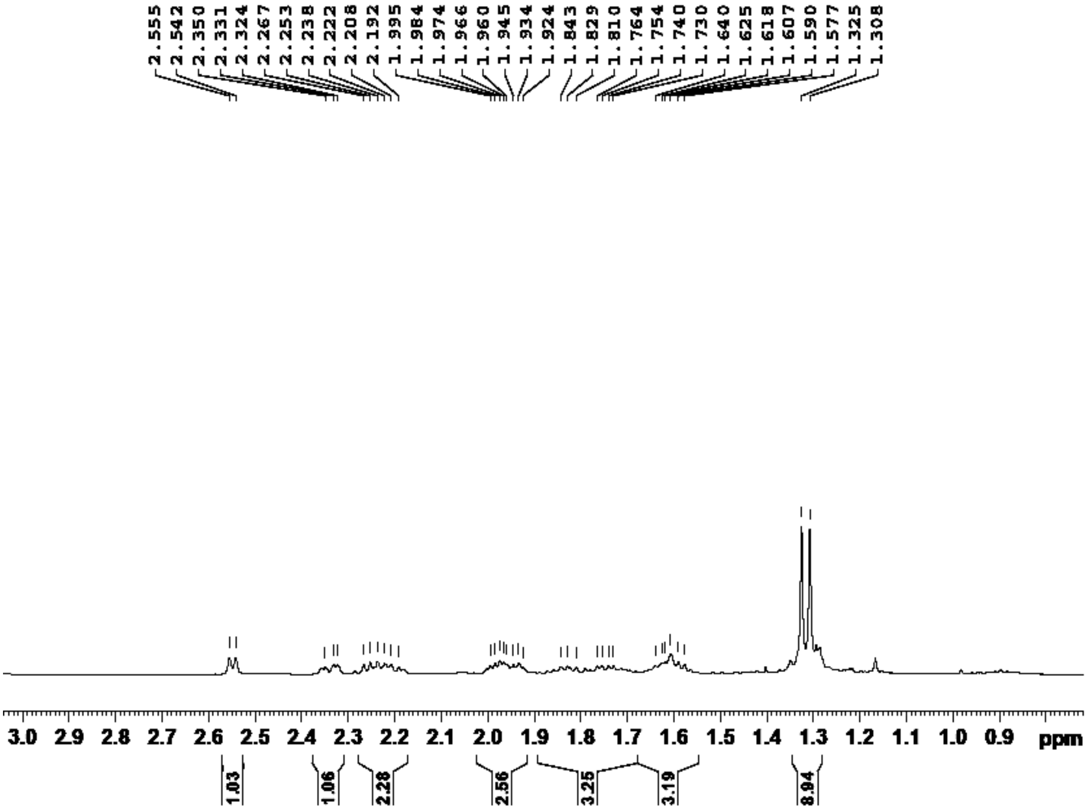

S3. Enlarged ^{1}H NMR (500 MHz, CD_3OD) spectrum 2 for **1**.

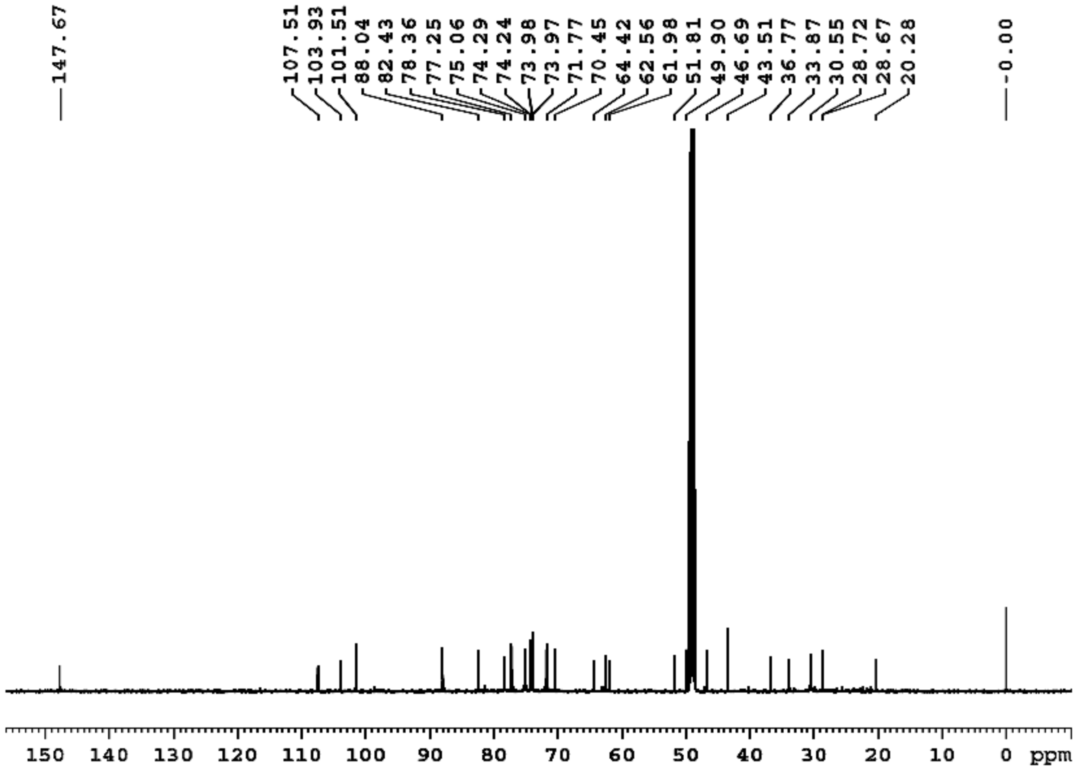

S4. ^{13}C NMR (125 MHz, CD_3OD) spectrum for **1**.

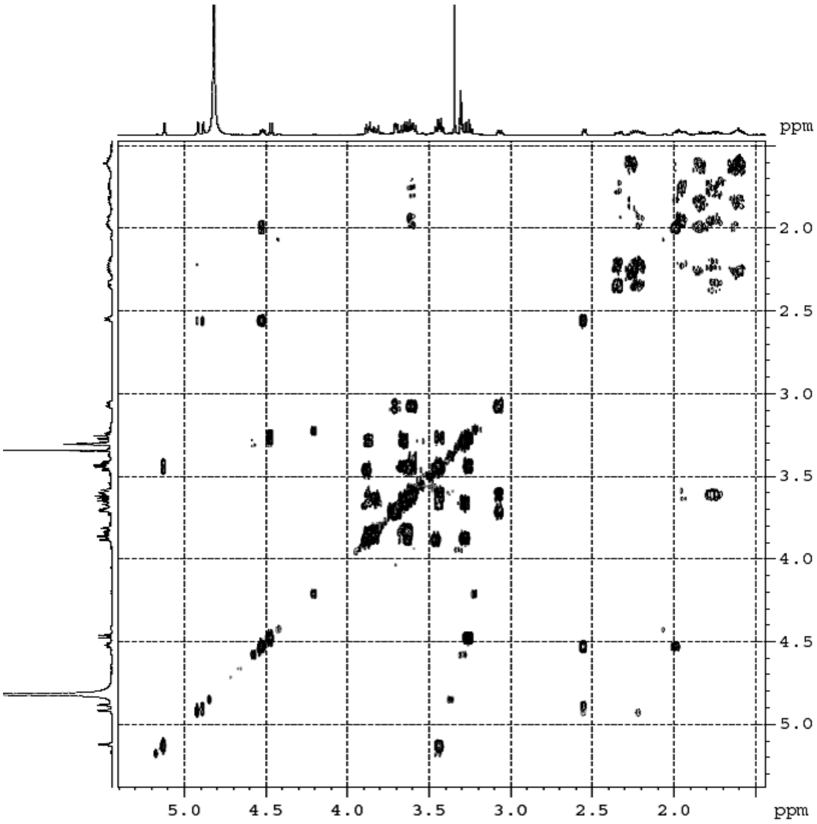

S5. ^1H ^1H COSY (CD_3OD) spectrum for **1**.

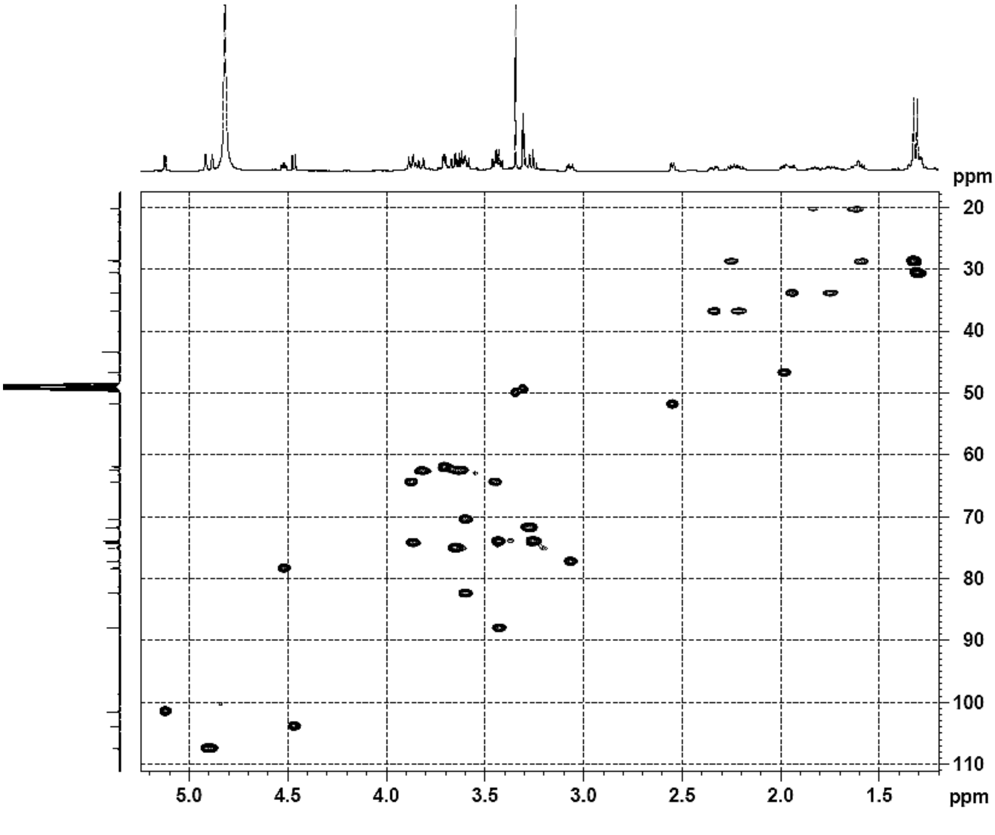

S6. HSQC (CD_3OD) spectrum for **1**.

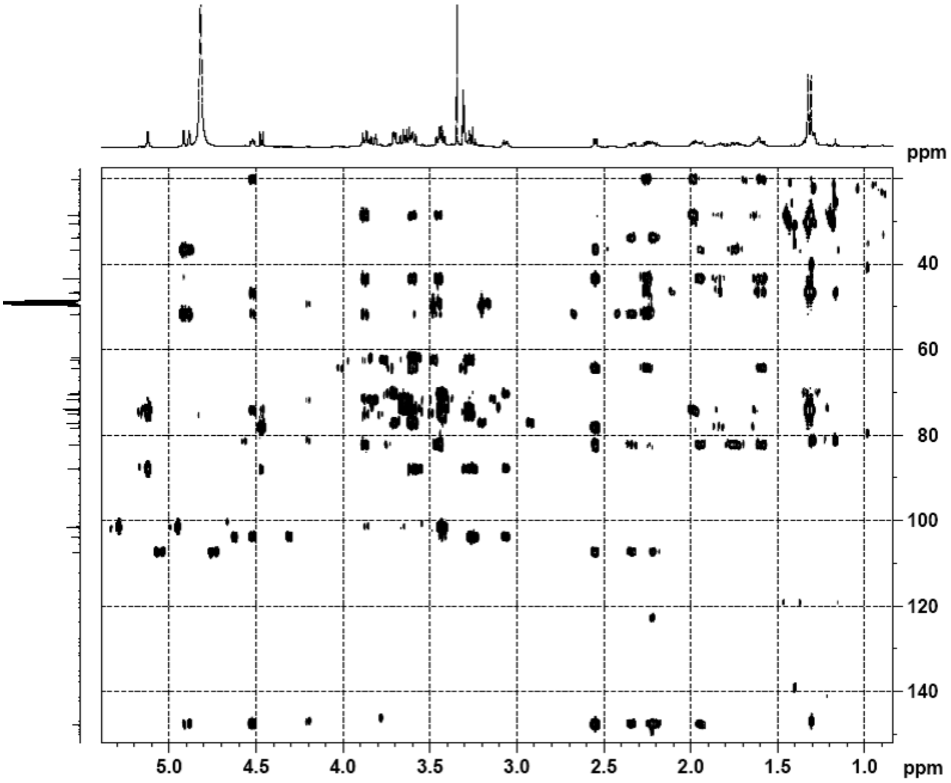

S7. HMBC (CD3OD) spectrum for **1**.

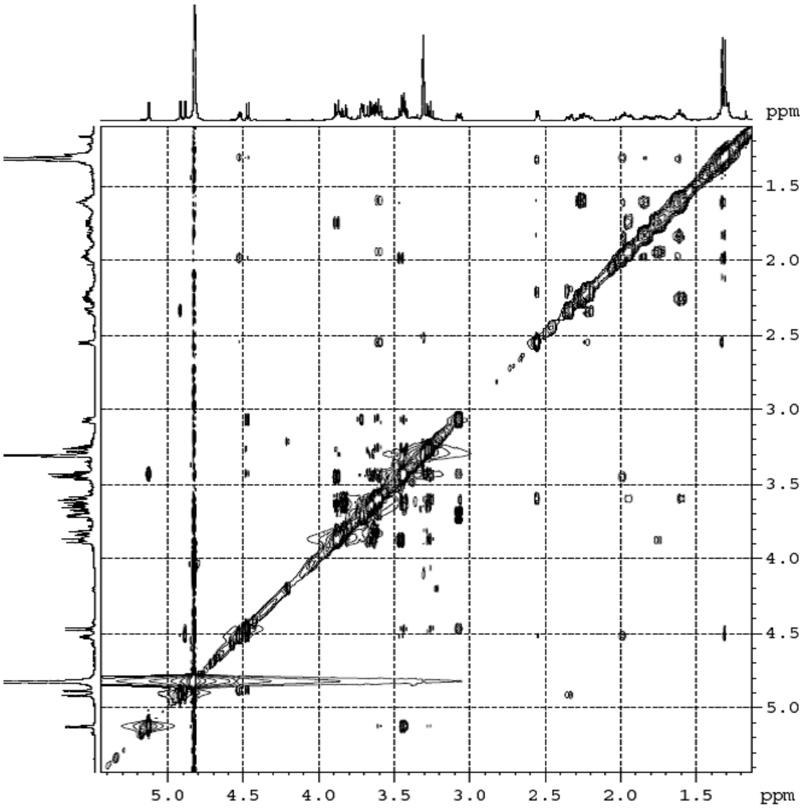

S8. NOESY (CD3OD) spectrum for **1**.

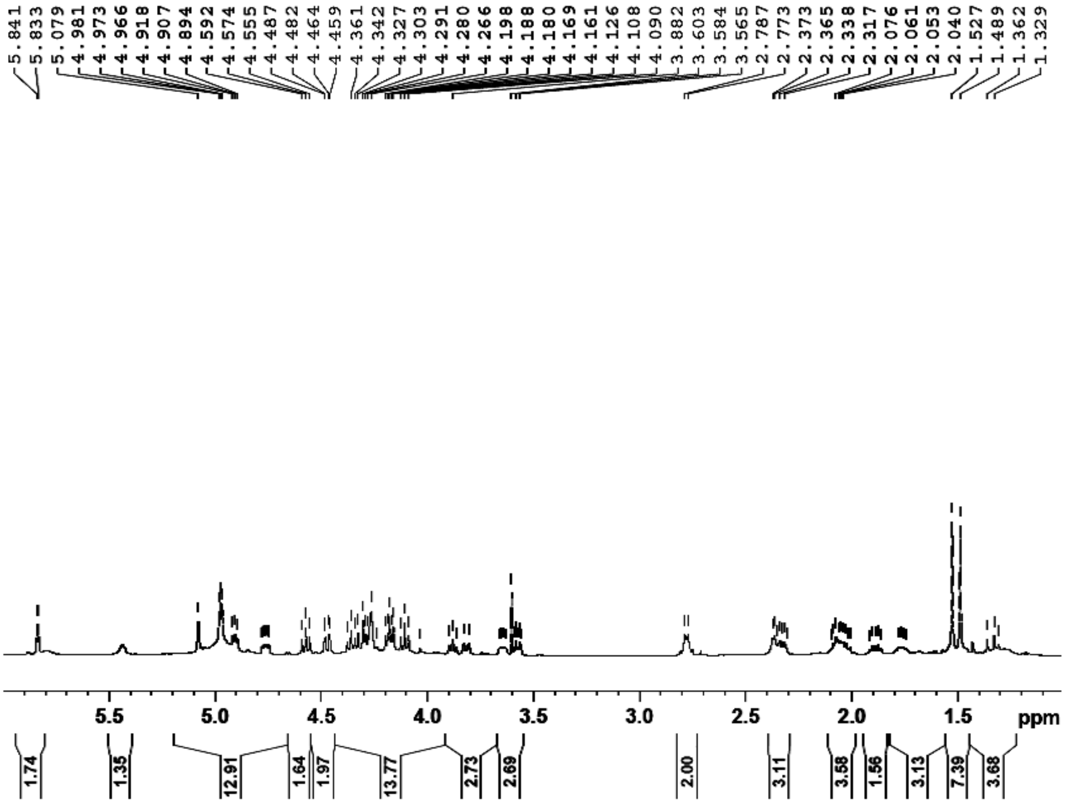

S9. ^{1}H NMR (500 MHz, C_5D_5N) spectrum for **1**.

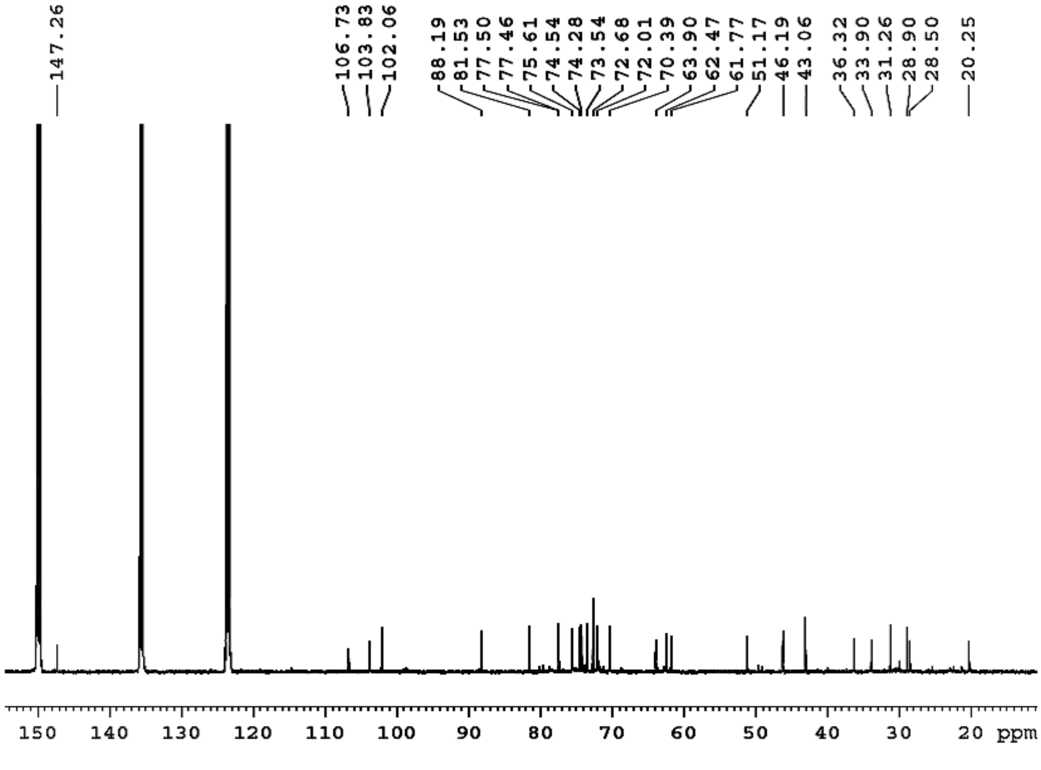

S10. ^{13}C NMR (125 MHz, C_5D_5N) spectrum for **1**.

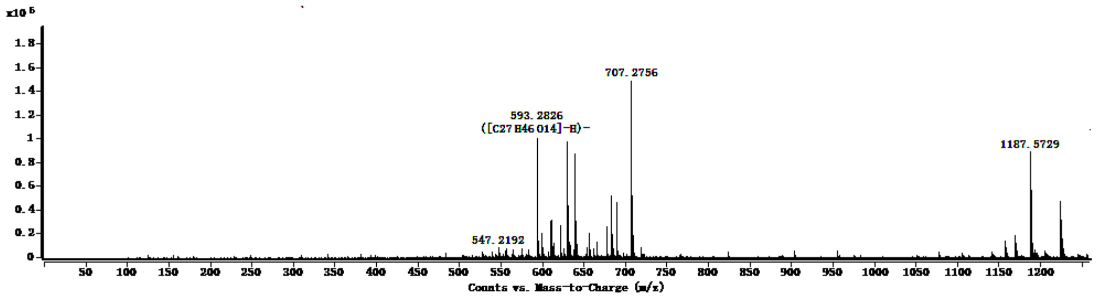

S11. HRESI-TOF-MS spectrum for **1**.

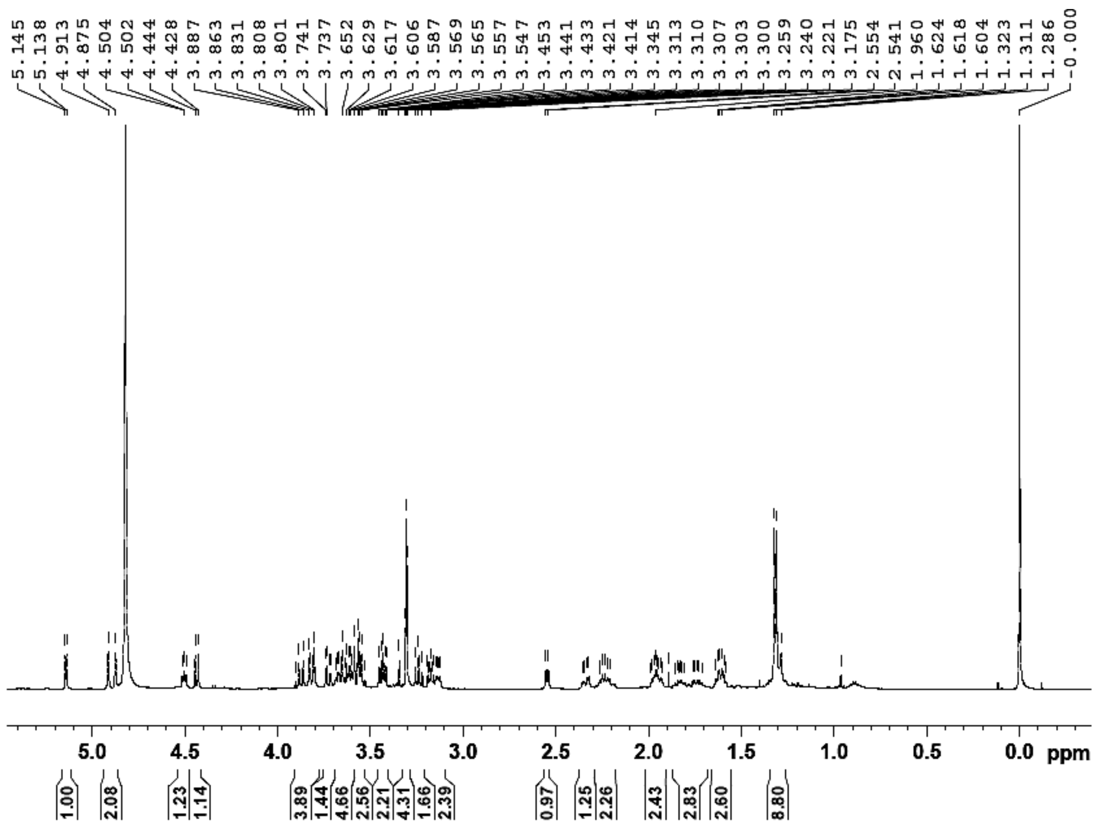

S12. ¹H NMR (500 MHz, CD_3OD) spectrum for **2**.

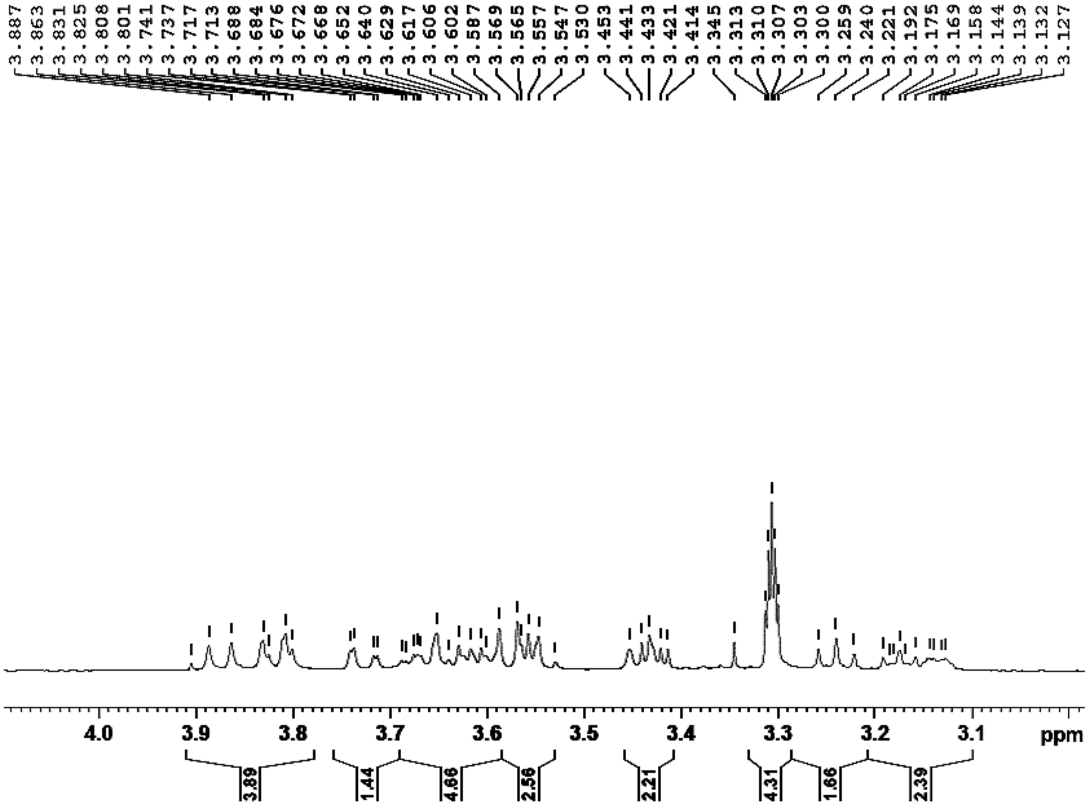

S13. Enlarged ¹H NMR (500 MHz, CD_3OD) spectrum 1 for **2**.

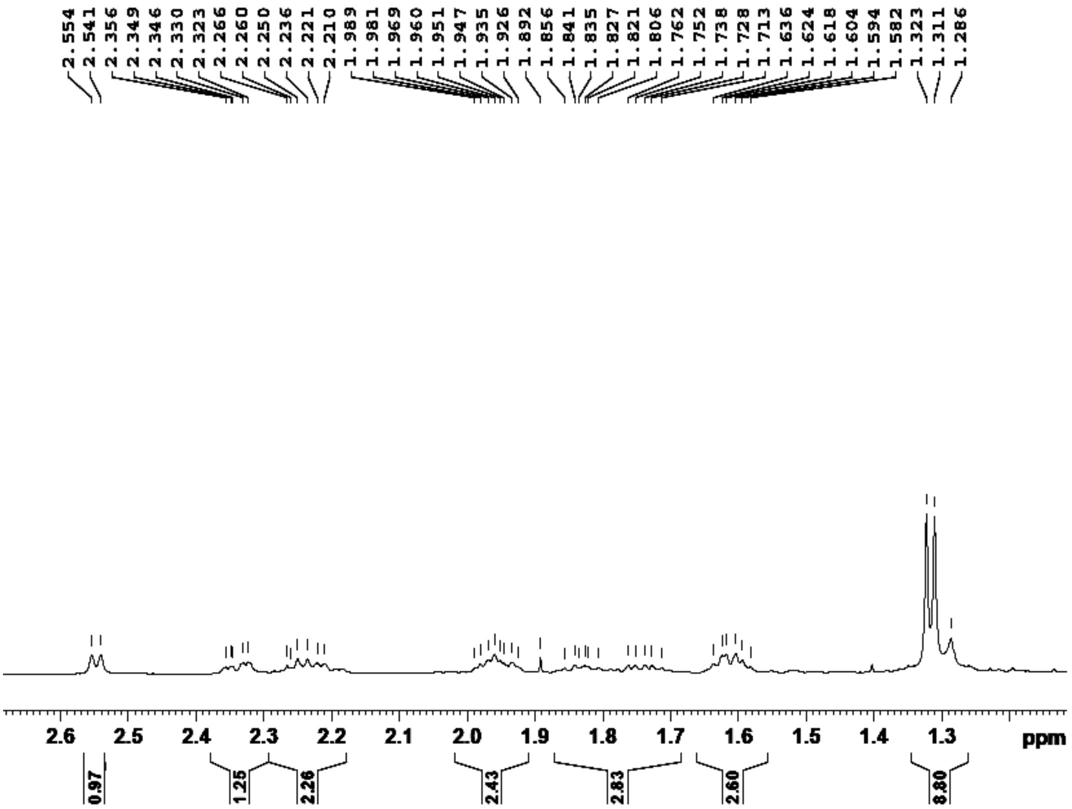

S14. Enlarged ^{1}H NMR (500 MHz, CD3OD) spectrum 2 for **2**.

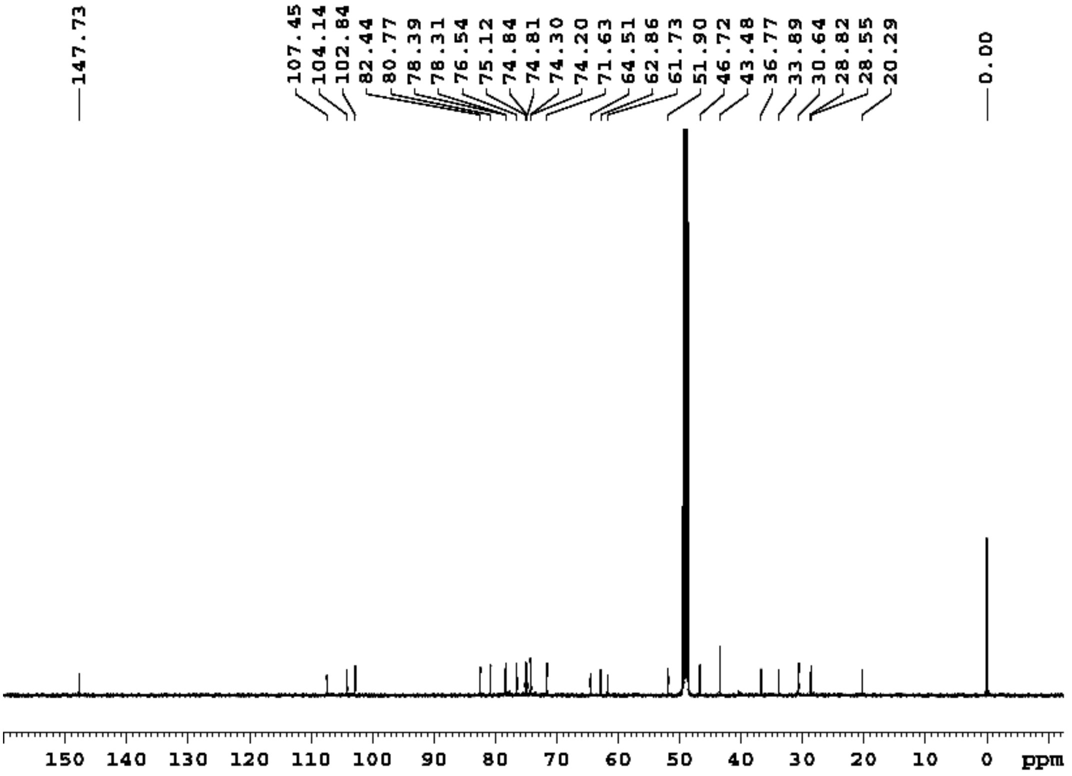

S15. ^{13}C NMR (125 MHz, CD3OD) spectrum for **2**.

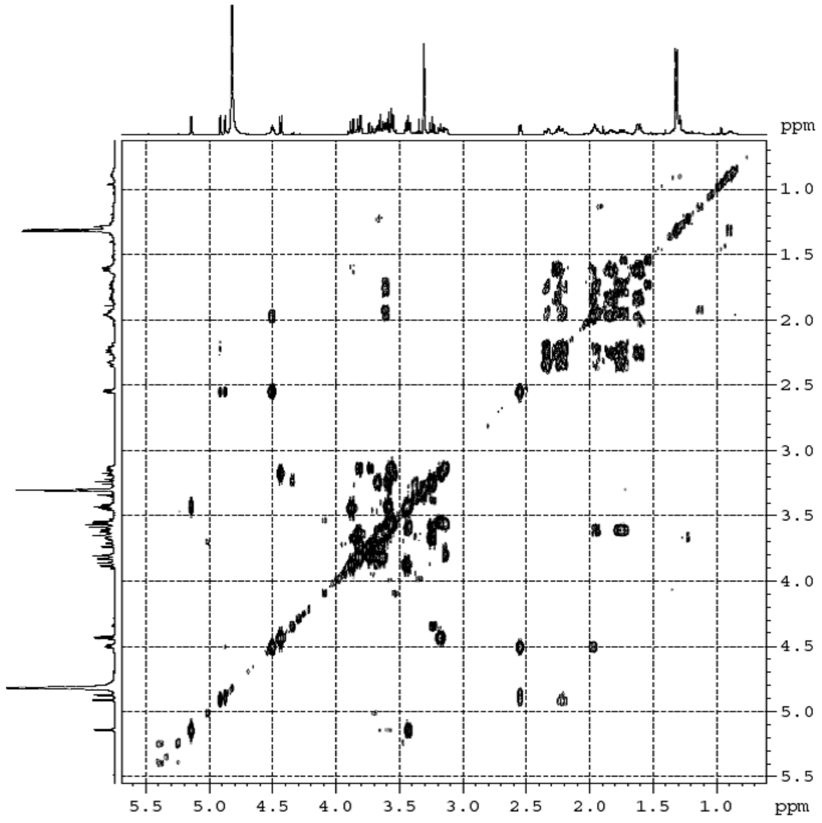

S16. ^{1}H ^{1}H COSY (CD_3OD) spectrum for **2**.

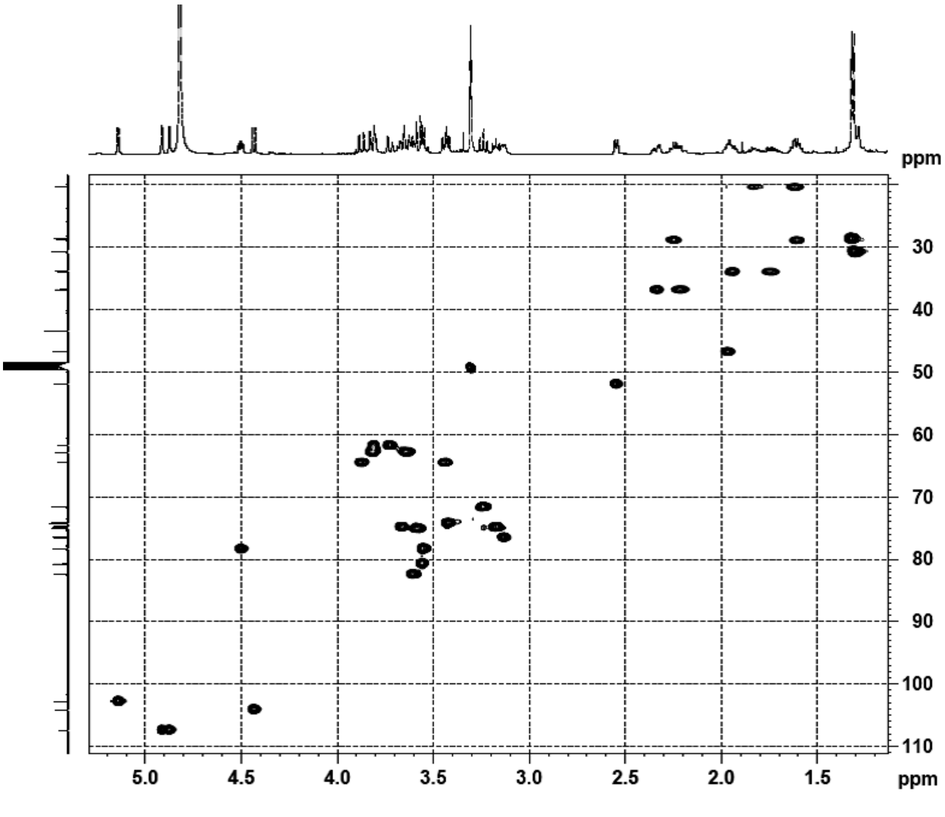

S17. HSQC (CD_3OD) spectrum for **2**.

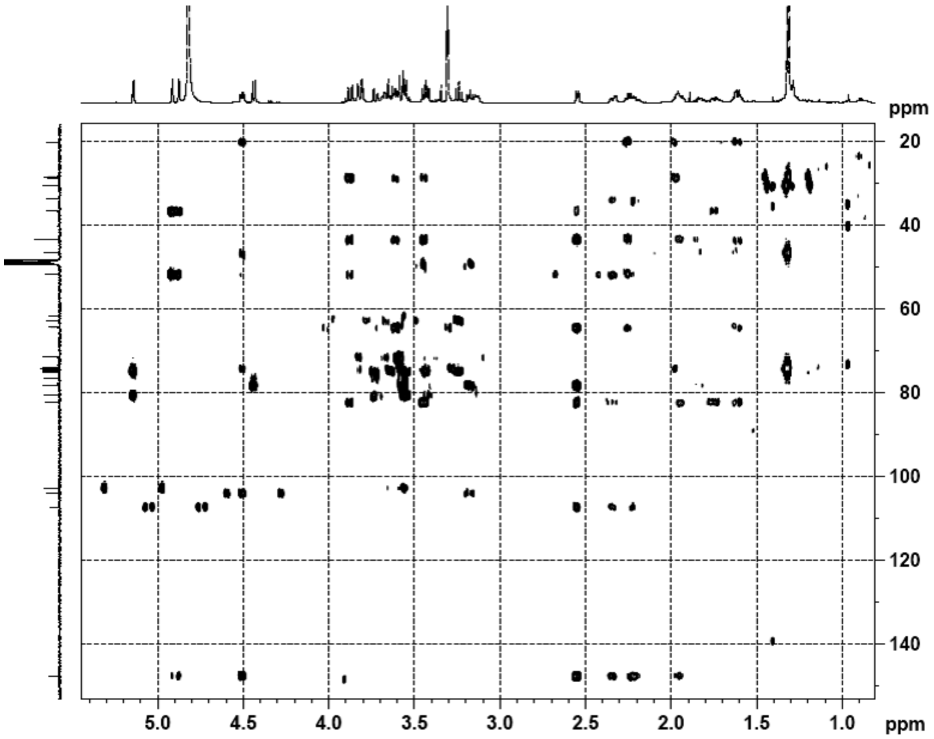

S18. HMBC (CD_3OD) spectrum for **2**.

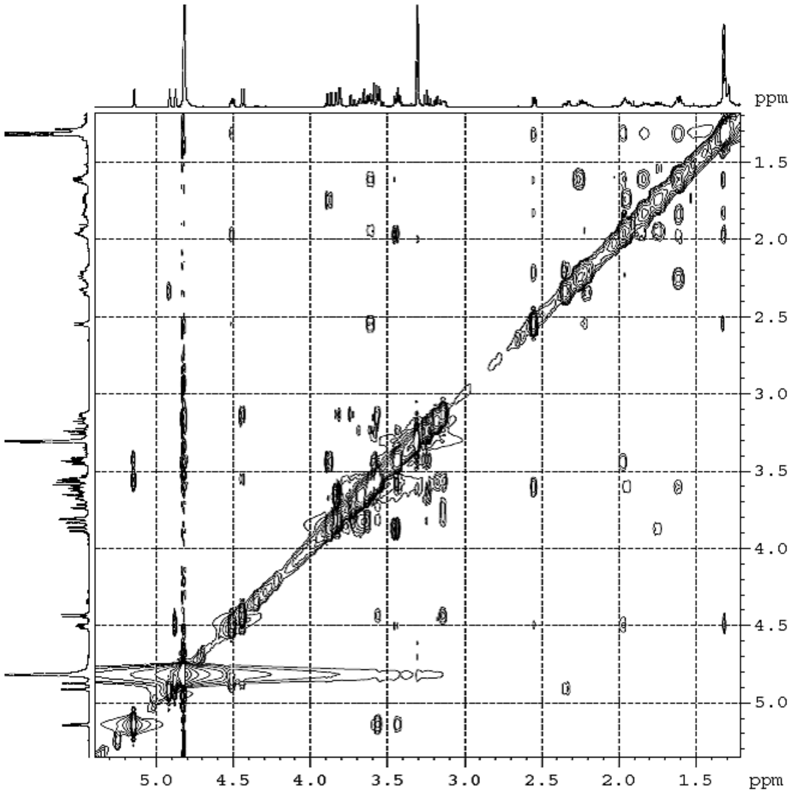

S19. NOESY (CD_3OD) spectrum for **2**.

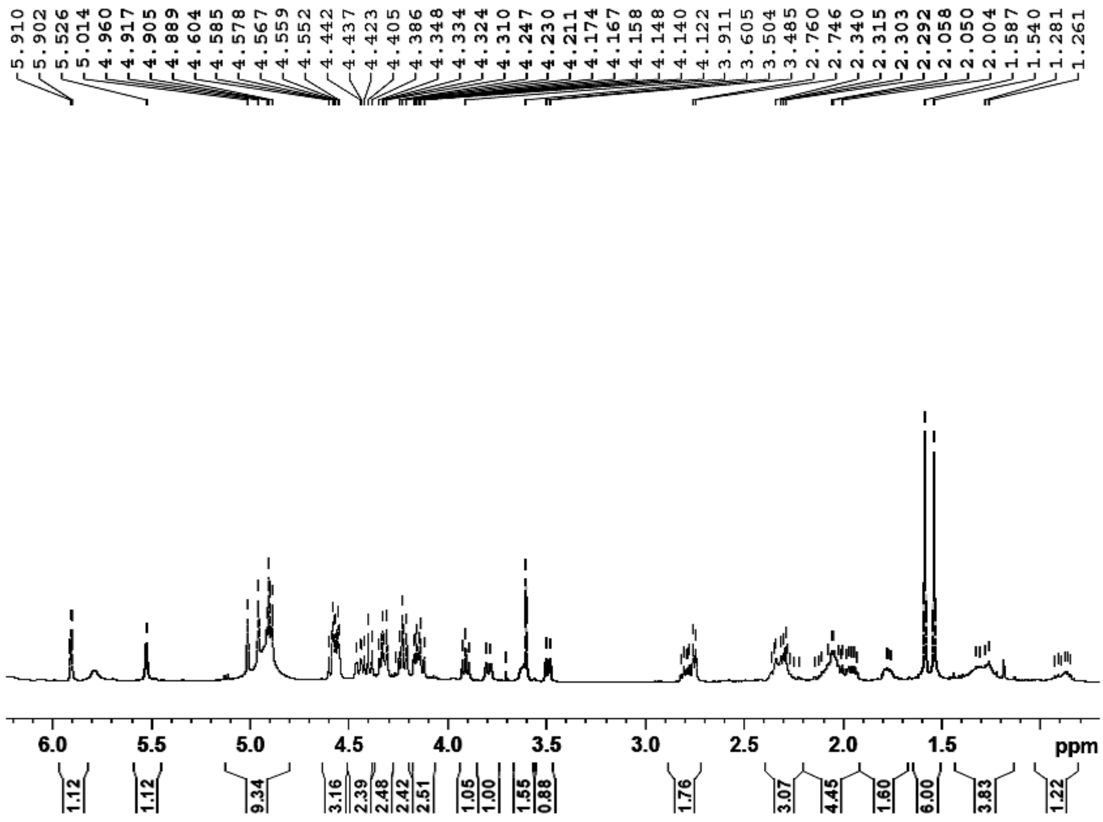

S20. ^{1}H NMR (500 MHz, C_5D_5N) spectrum for **2**.

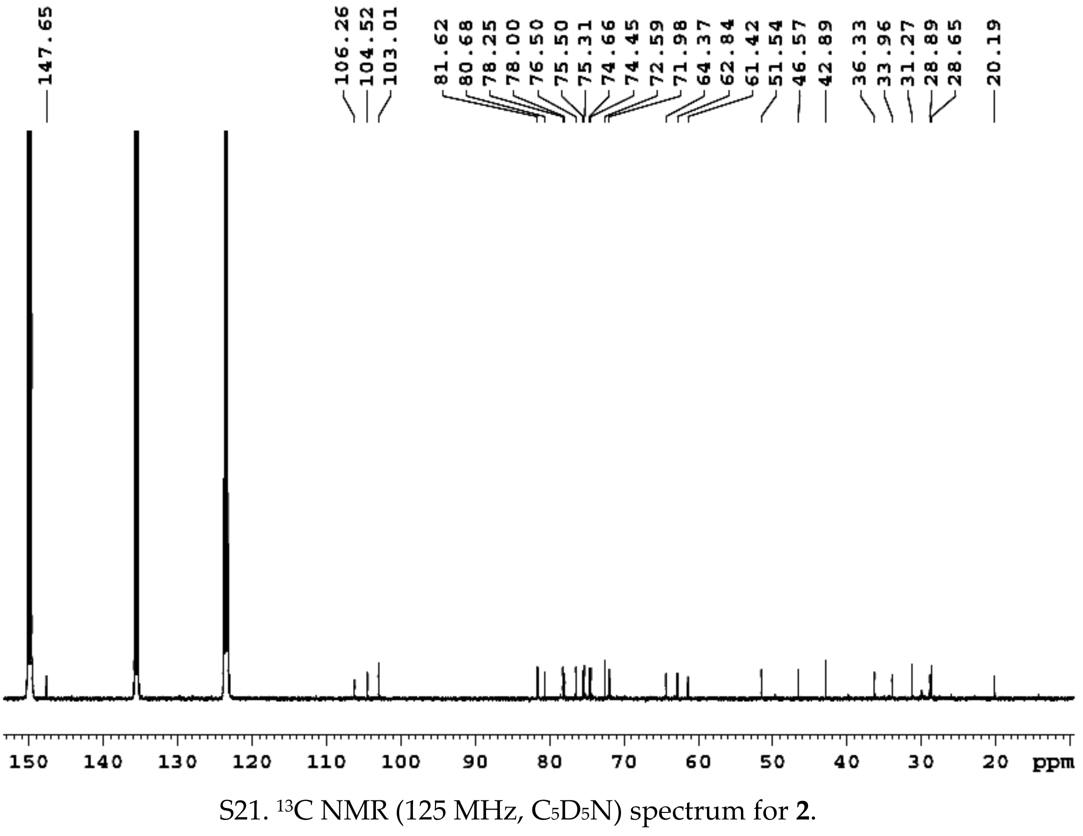

S21. ^{13}C NMR (125 MHz, C_5D_5N) spectrum for **2**.

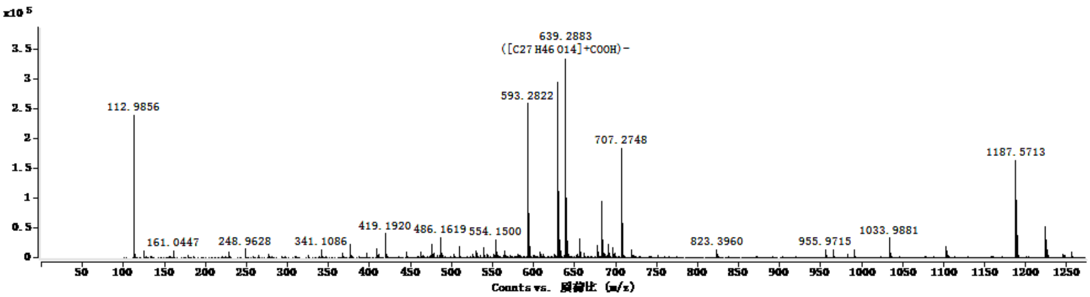

S22. HRESI-TOF-MS spectrum for **2**.

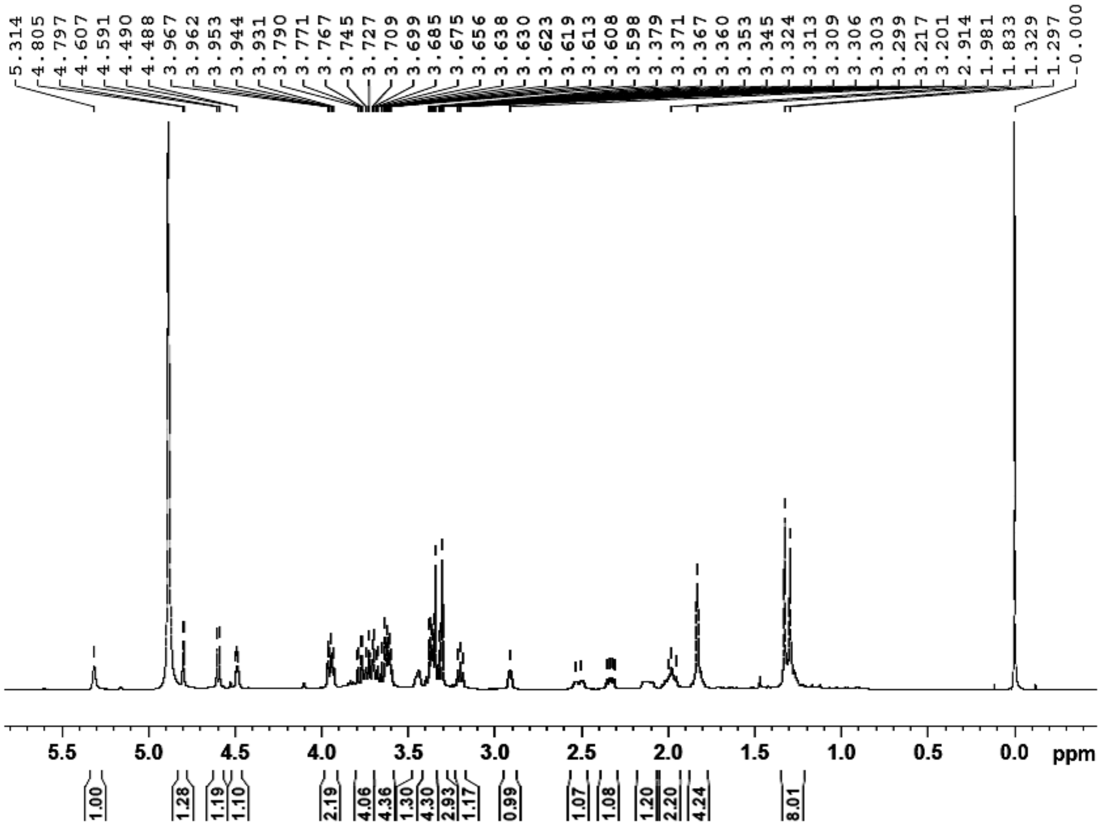

S23. ^{1}H NMR (500 MHz, CD_3OD) spectrum for **3**.

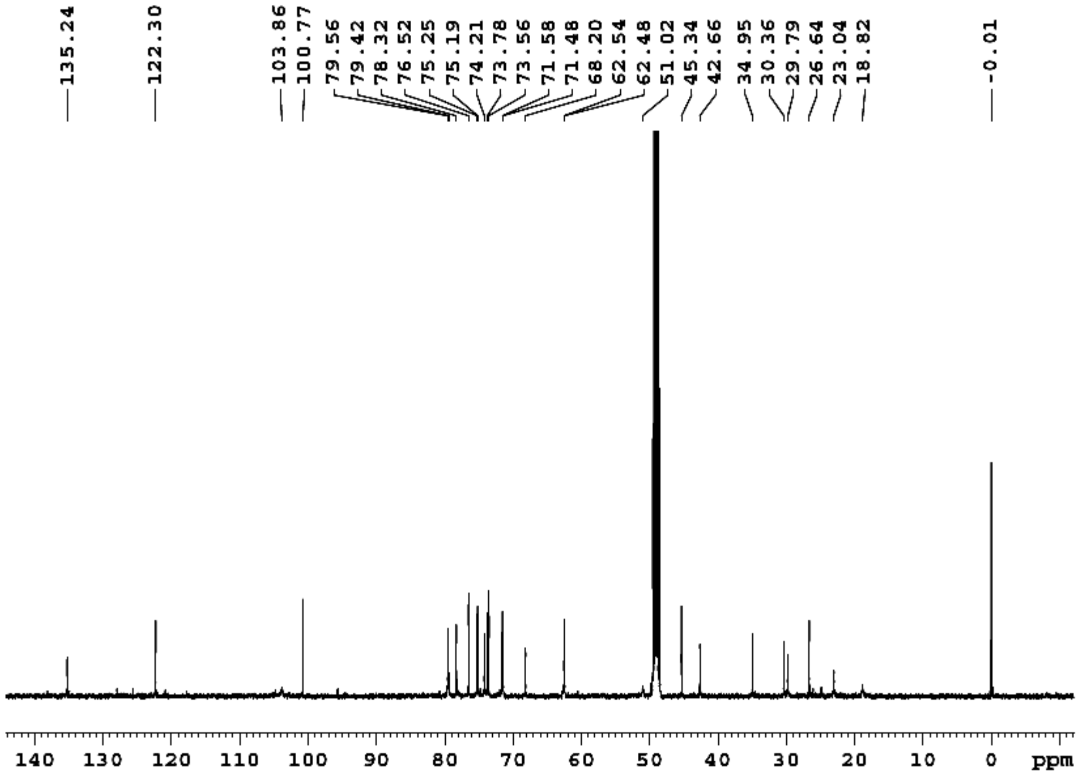

S24. ^{13}C NMR (125 MHz, CD_3OD) spectrum for **3**.

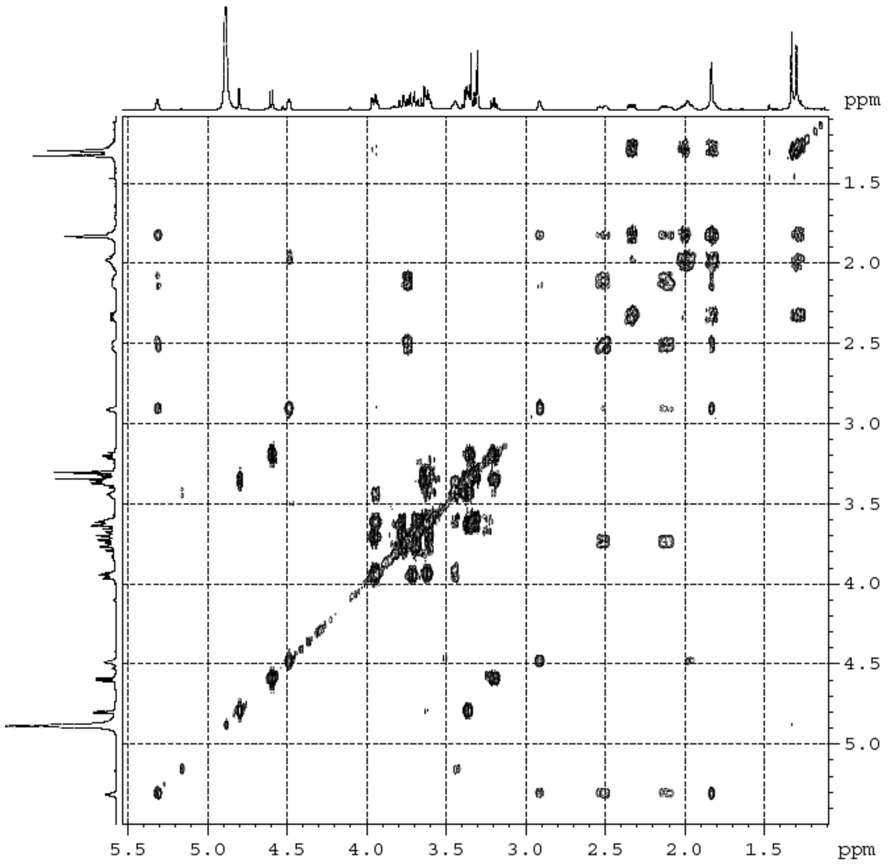

S25. ^{1}H ^{1}H COSY (CD_3OD) spectrum for **3**.

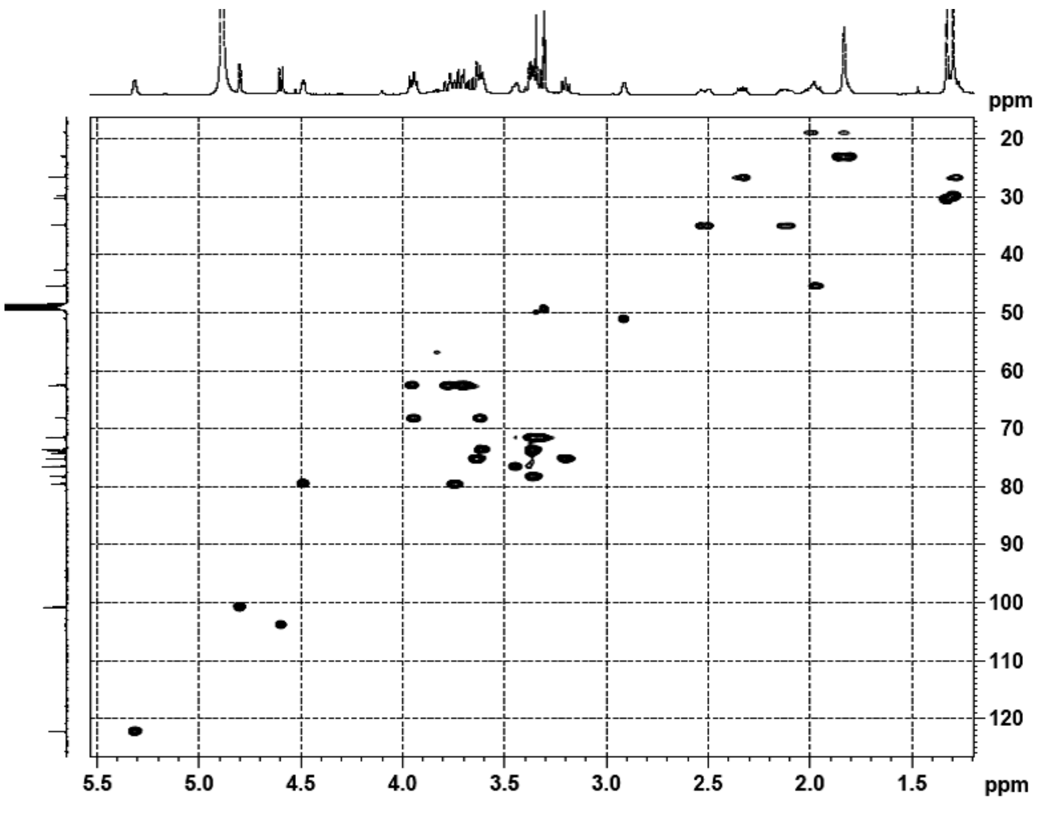

S26. HSQC (CD_3OD) spectrum for **3**.

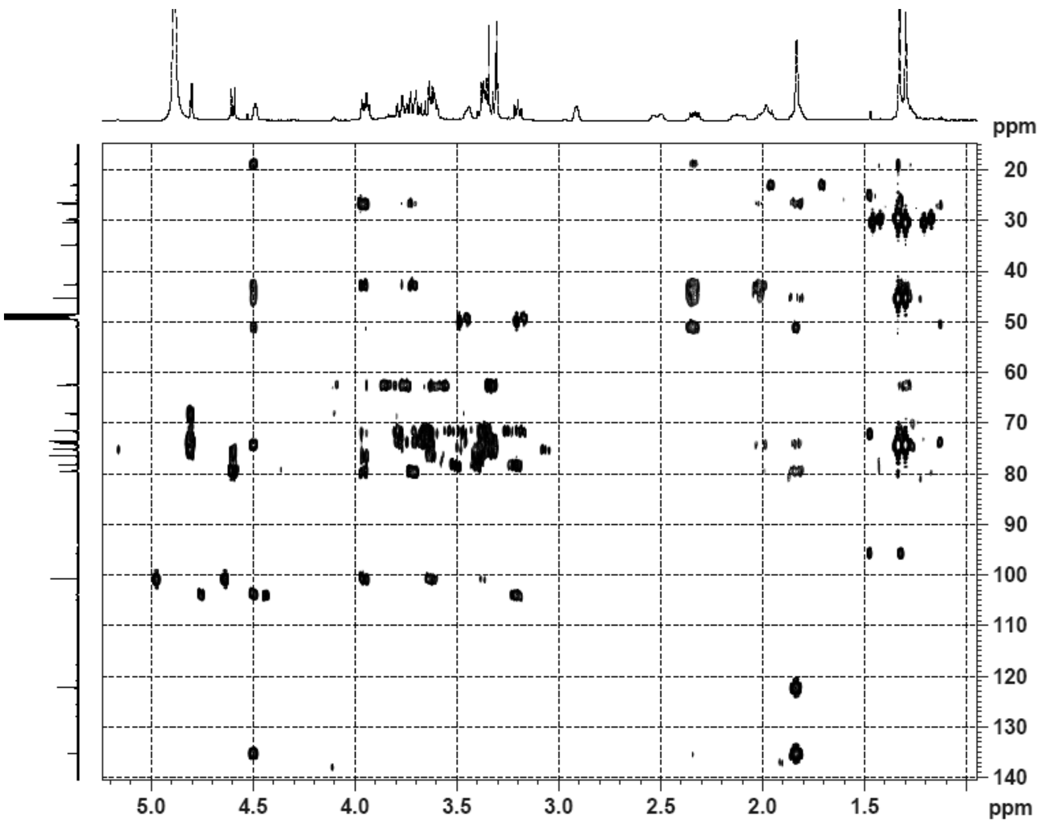

S27. HMBC (CD3OD) spectrum for **3**.

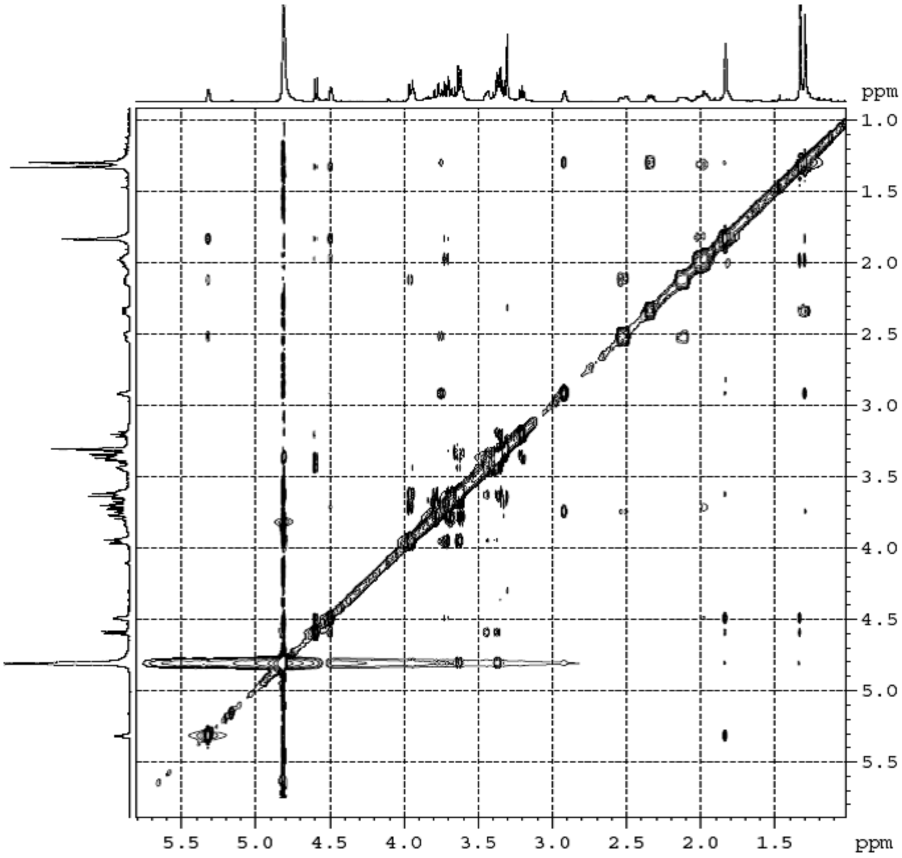

S28. NOESY (CD3OD) spectrum for **3**.

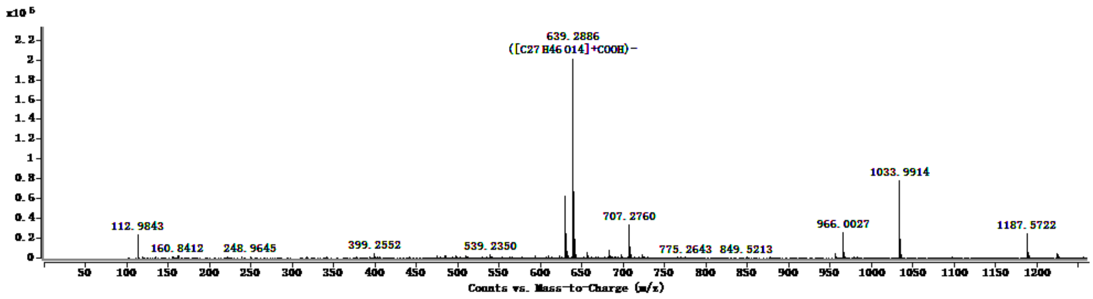

S29. HRESI-TOF-MS spectrum for **3**.

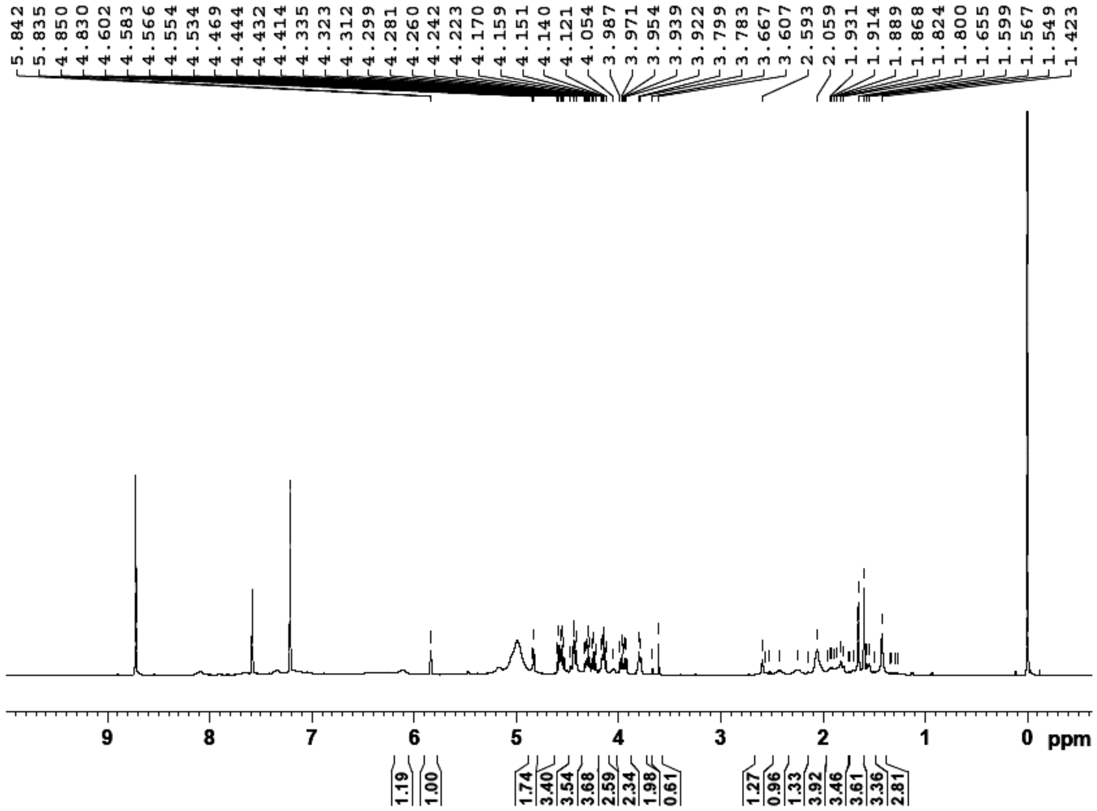

S30. ^{1}H NMR (500 MHz, C_5D_5N) spectrum for **4**.

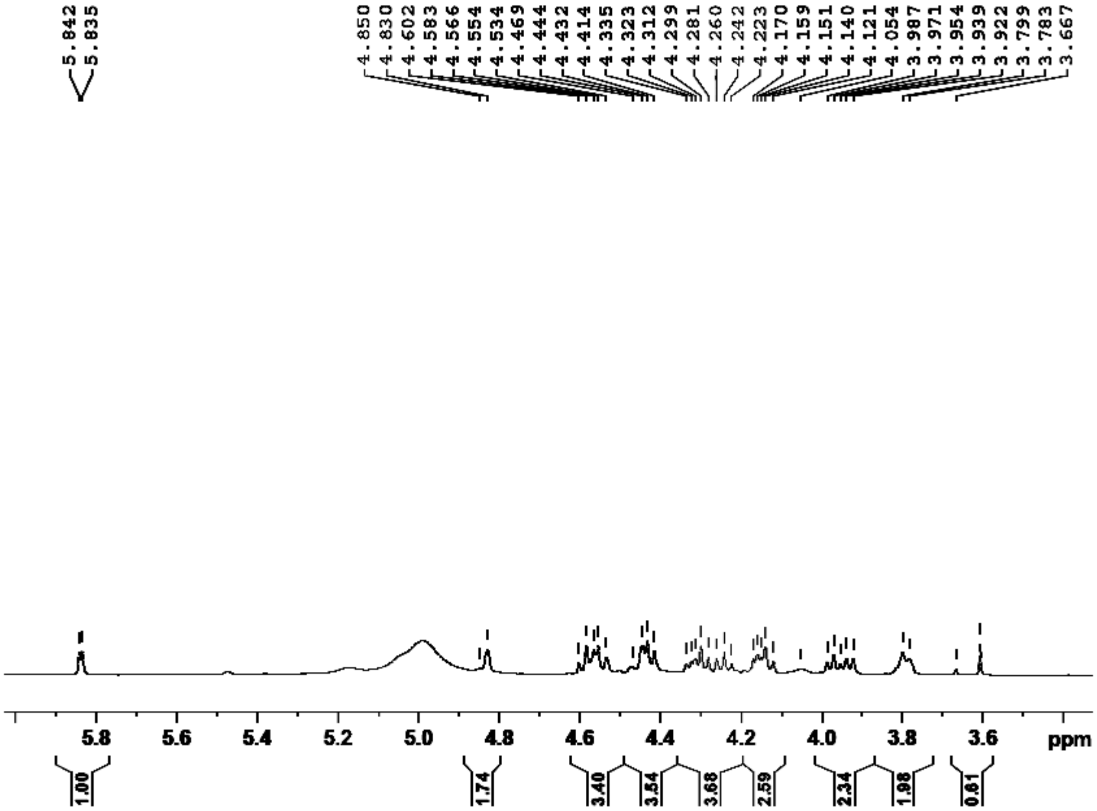

S31. Enlarged ^{1}H NMR (500 MHz, C_5D_5N) spectrum 1 for **4**.

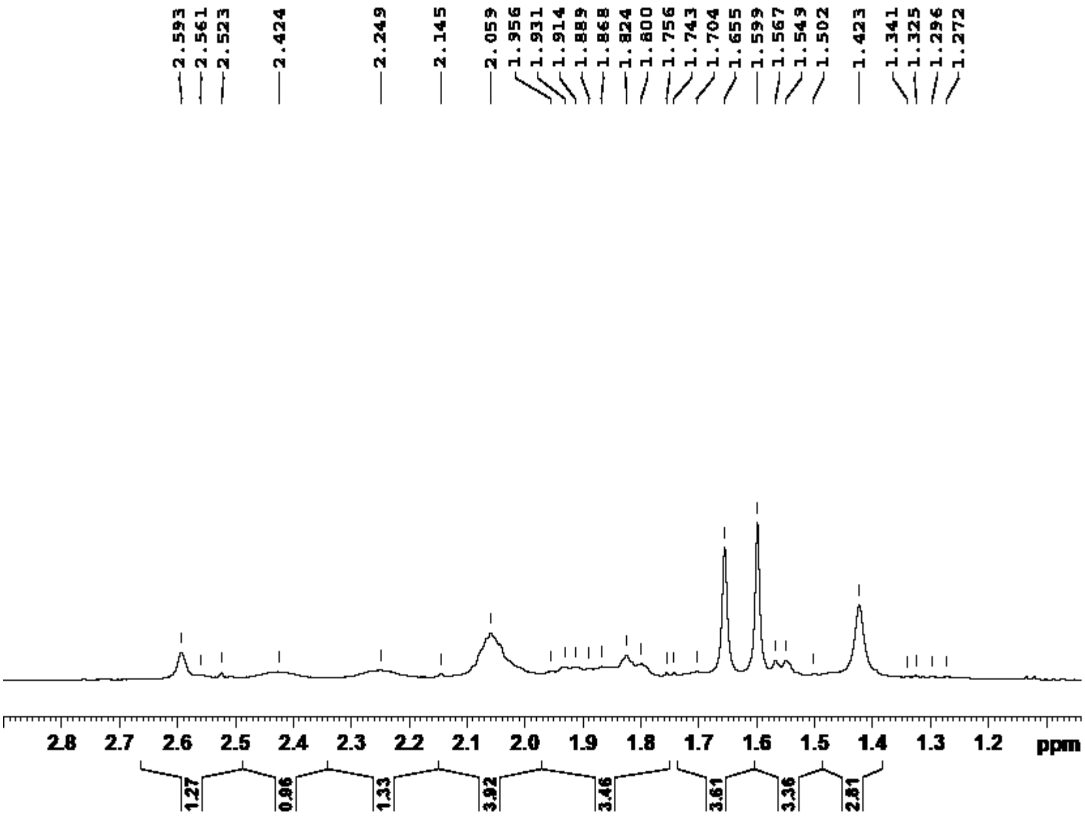

S32. Enlarged ^{1}H NMR (500 MHz, C_5D_5N) spectrum 2 for **4**.

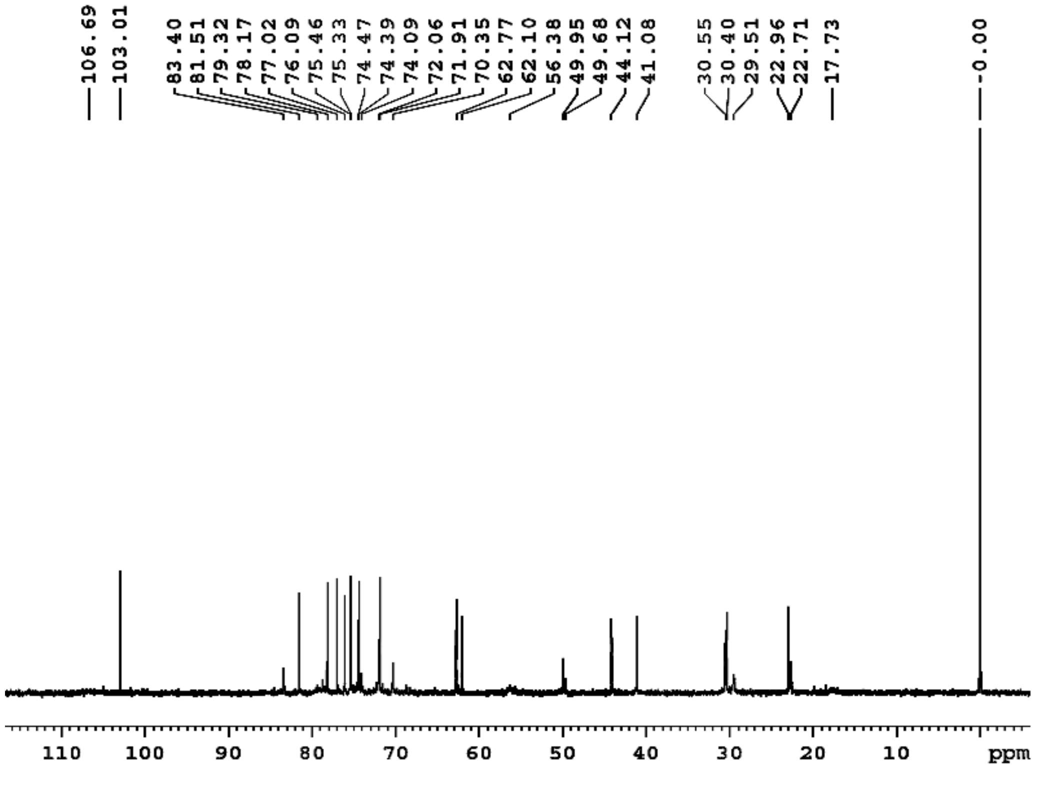

S33. ^{13}C NMR (125 MHz, C_5D_5N) spectrum for **4**.

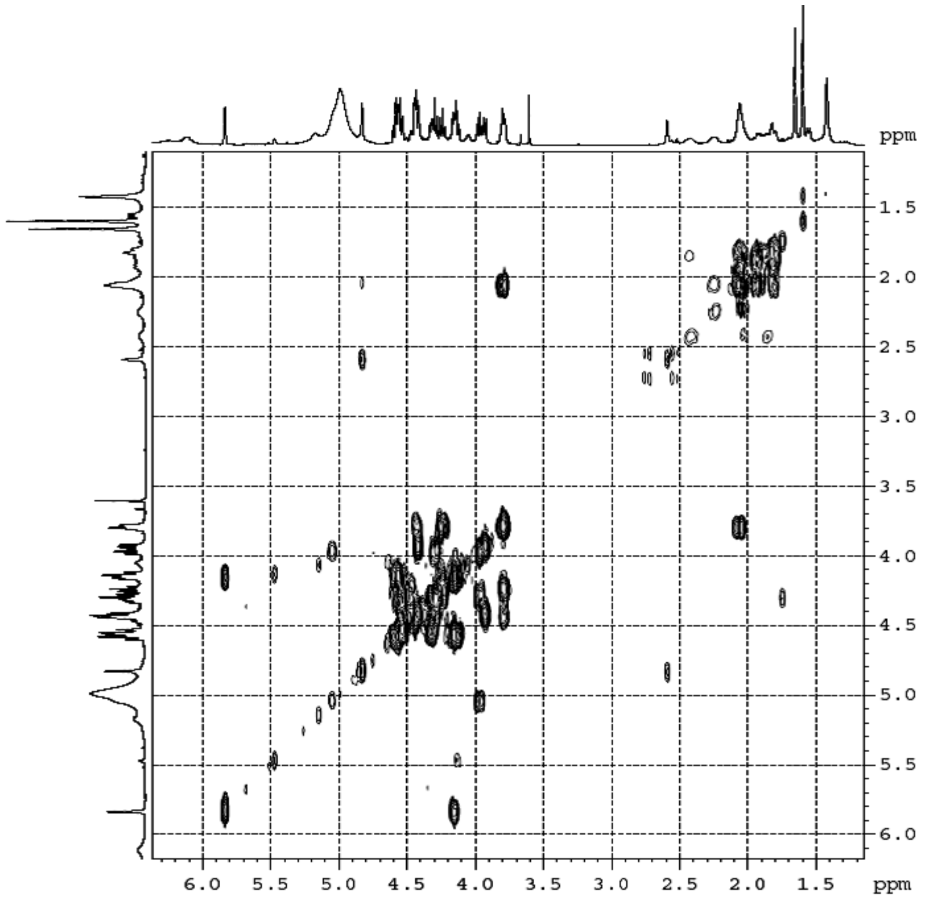

S34. ^{1}H ^{1}H COSY (C_5D_5N) spectrum for **4**.

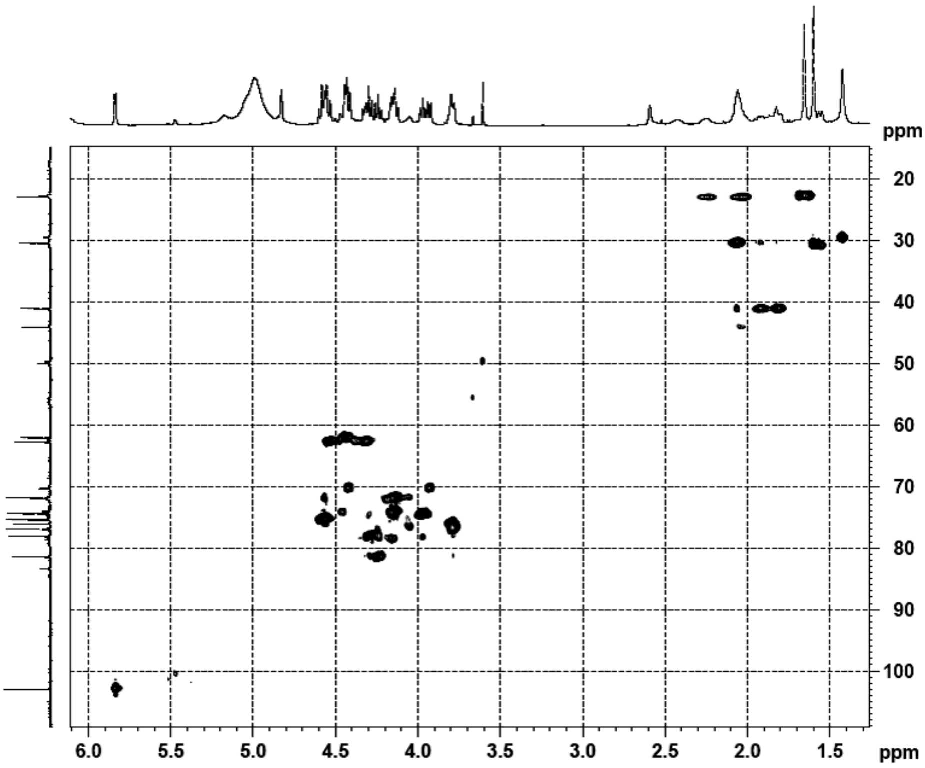

S35. HSQC (C_5D_5N) spectrum for **4**.

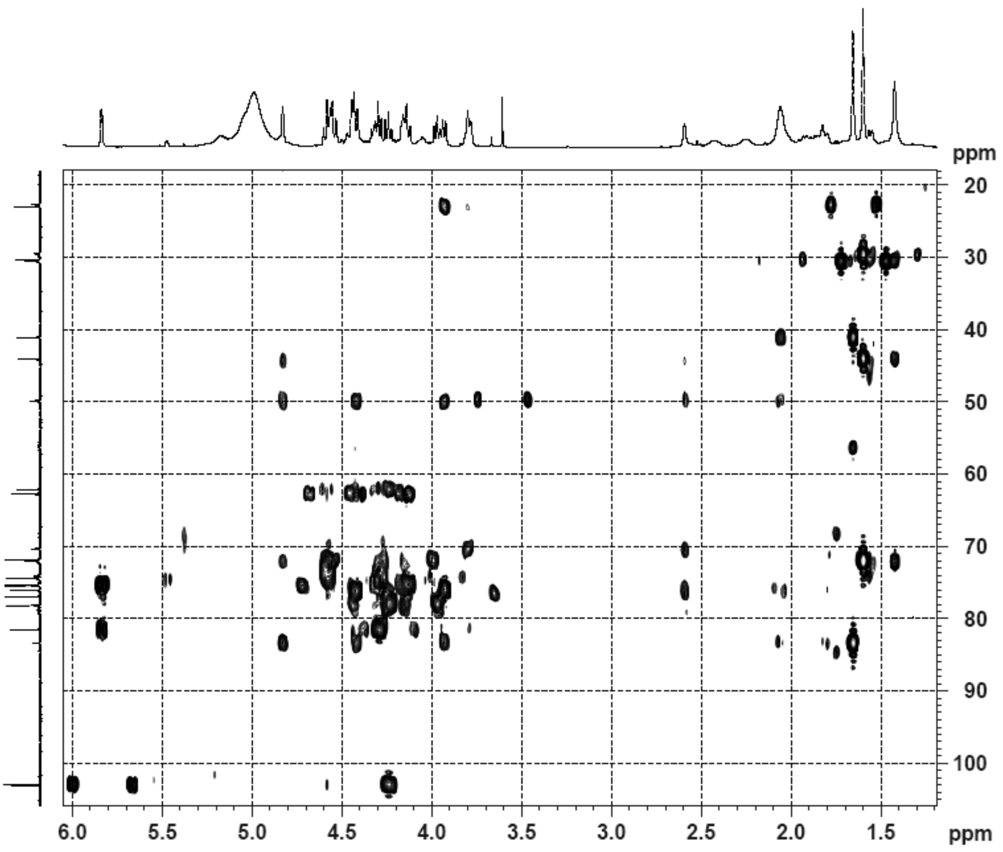

S36. HMBC (C_5D_5N) spectrum for **4**.

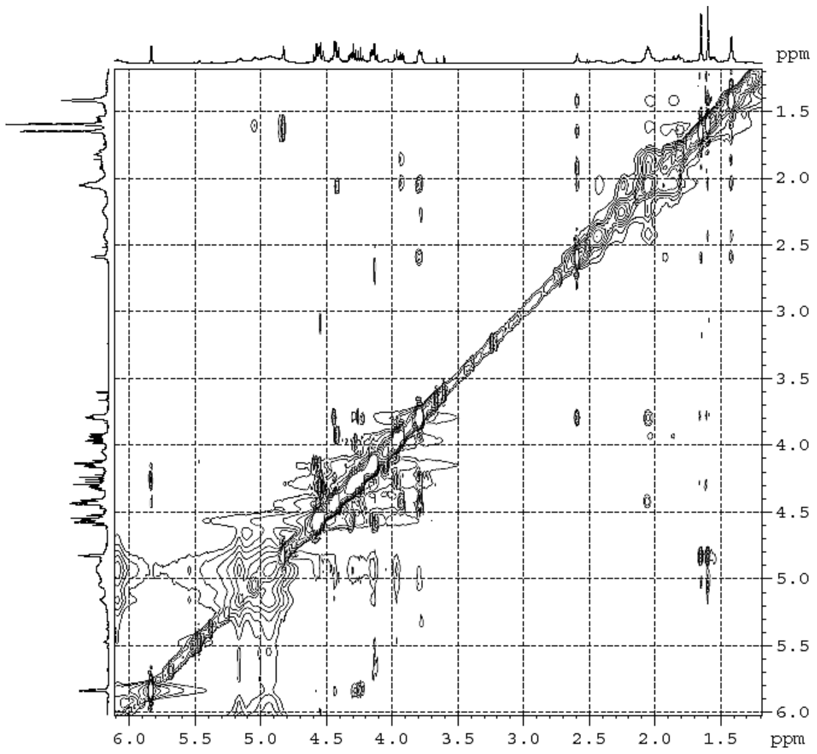

S37. NOESY (C_5D_5N) spectrum for **4**.

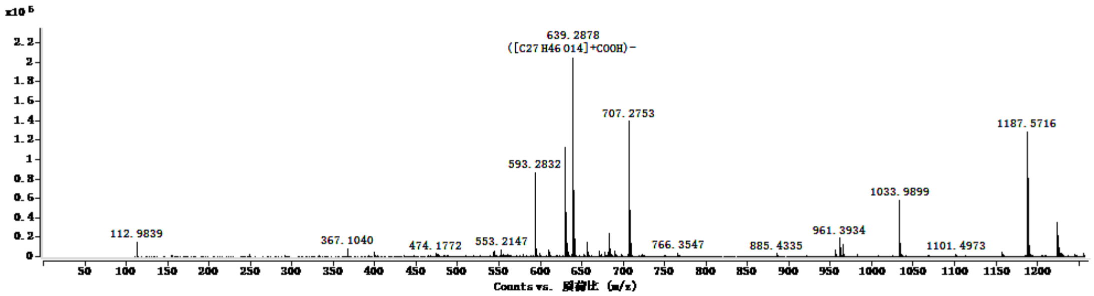

S38. HRESI-TOF-MS spectrum for **4**.

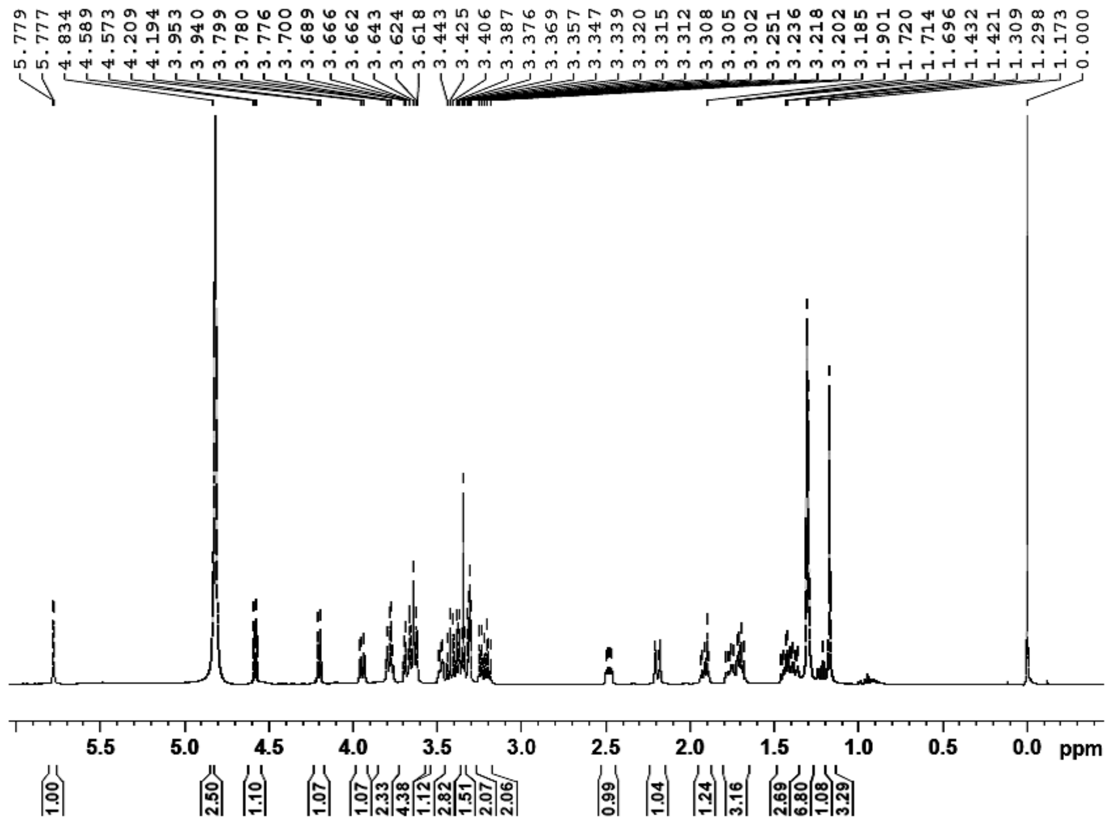

S39. ^{1}H NMR (500 MHz, CD_3OD) spectrum for **5**.

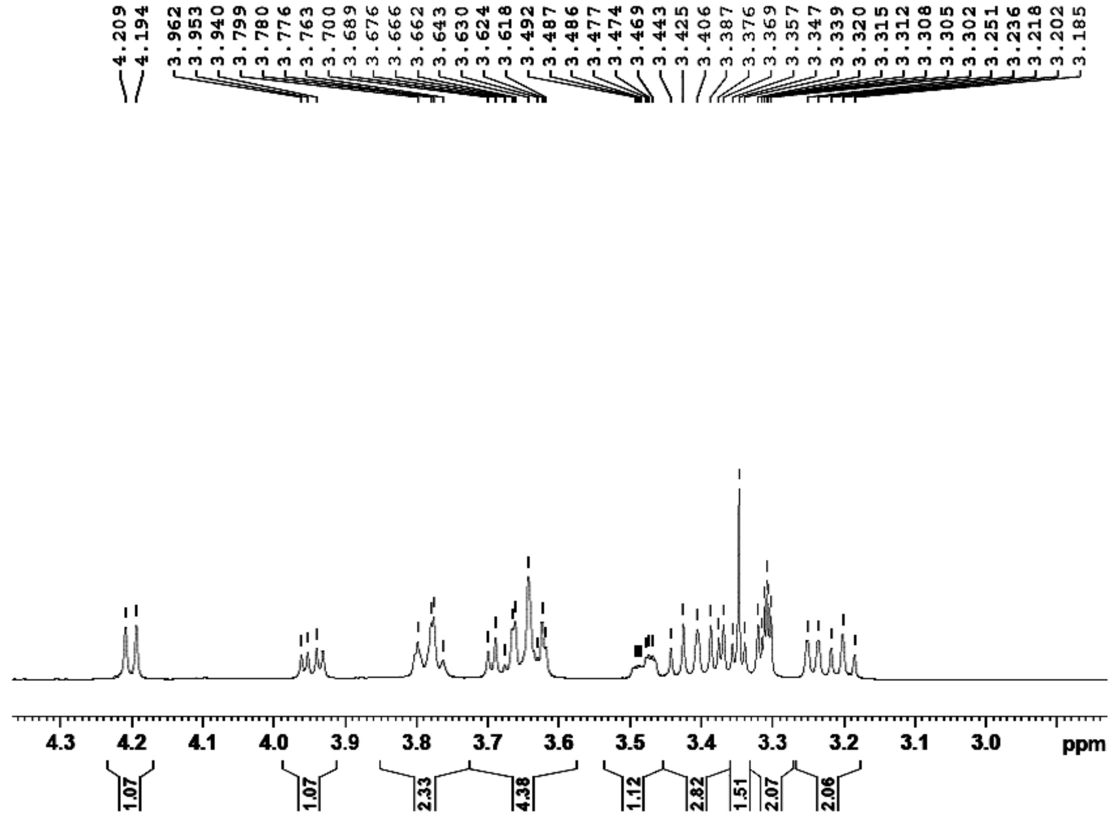

S40. Enlarged ^{1}H NMR (500 MHz, CD_3OD) spectrum 1 for **5**.

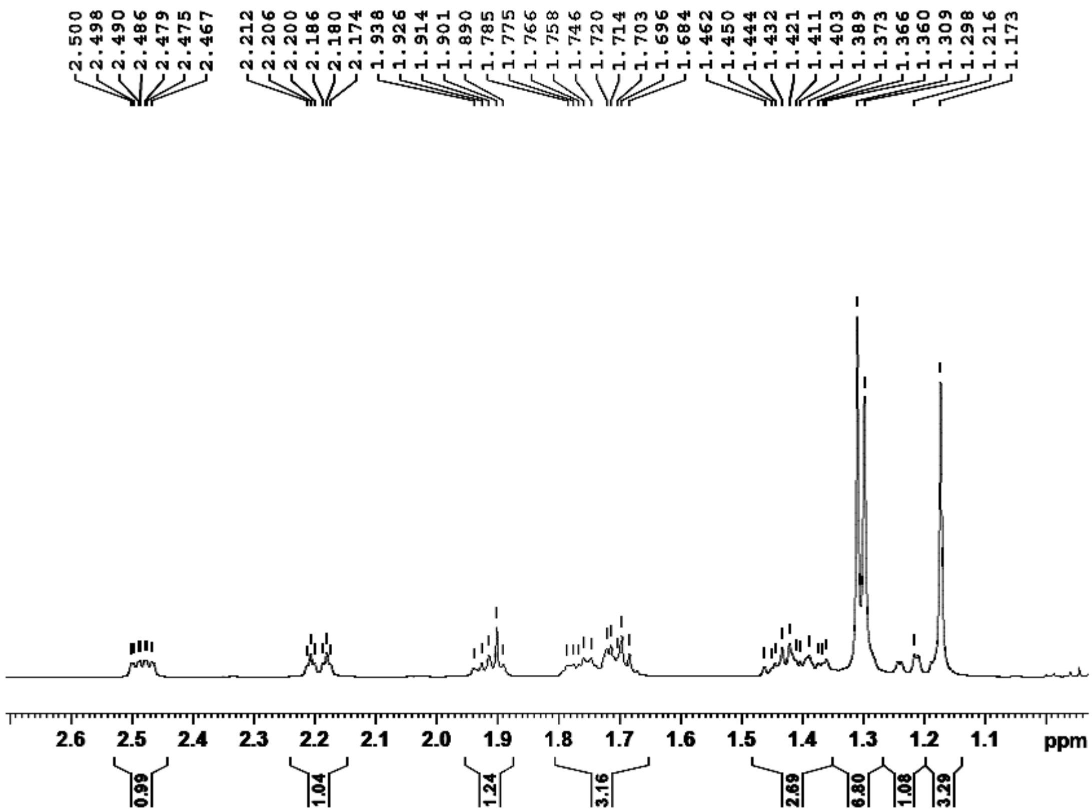

S41. Enlarged ^{1}H NMR (500 MHz, CD_3OD) spectrum 2 for **5**.

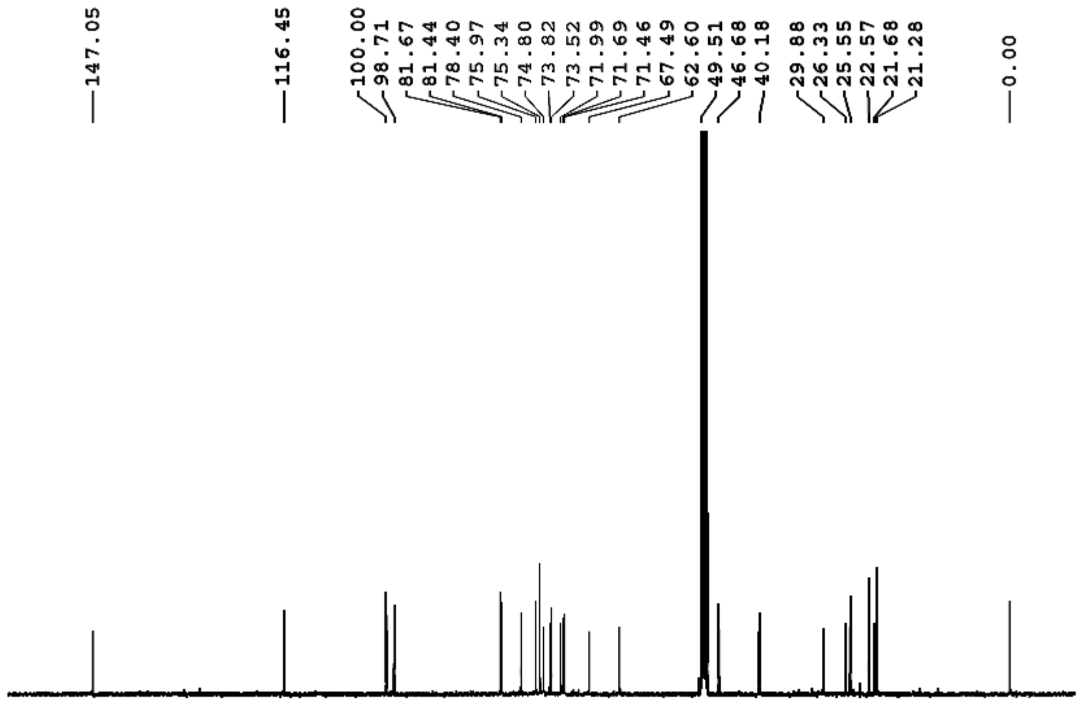

S42. ^{13}C NMR (125 MHz, CD_3OD) spectrum for **5**.

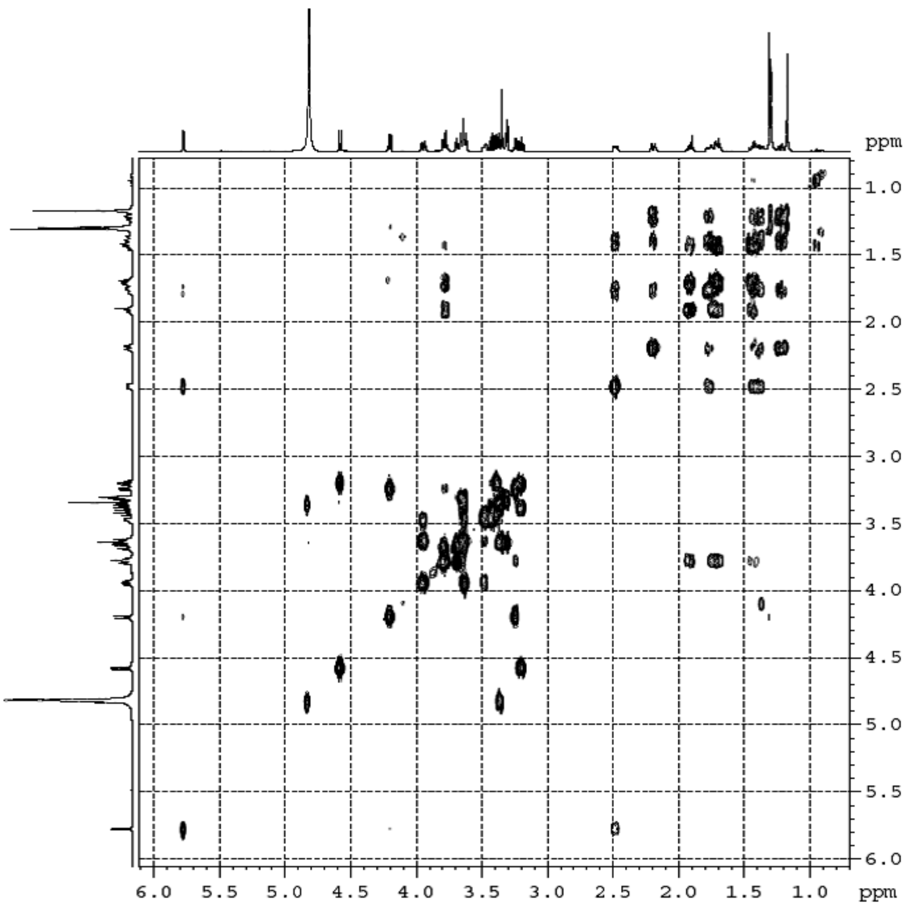

S43. ^{1}H ^{1}H COSY (CD_3OD) spectrum for **5**.

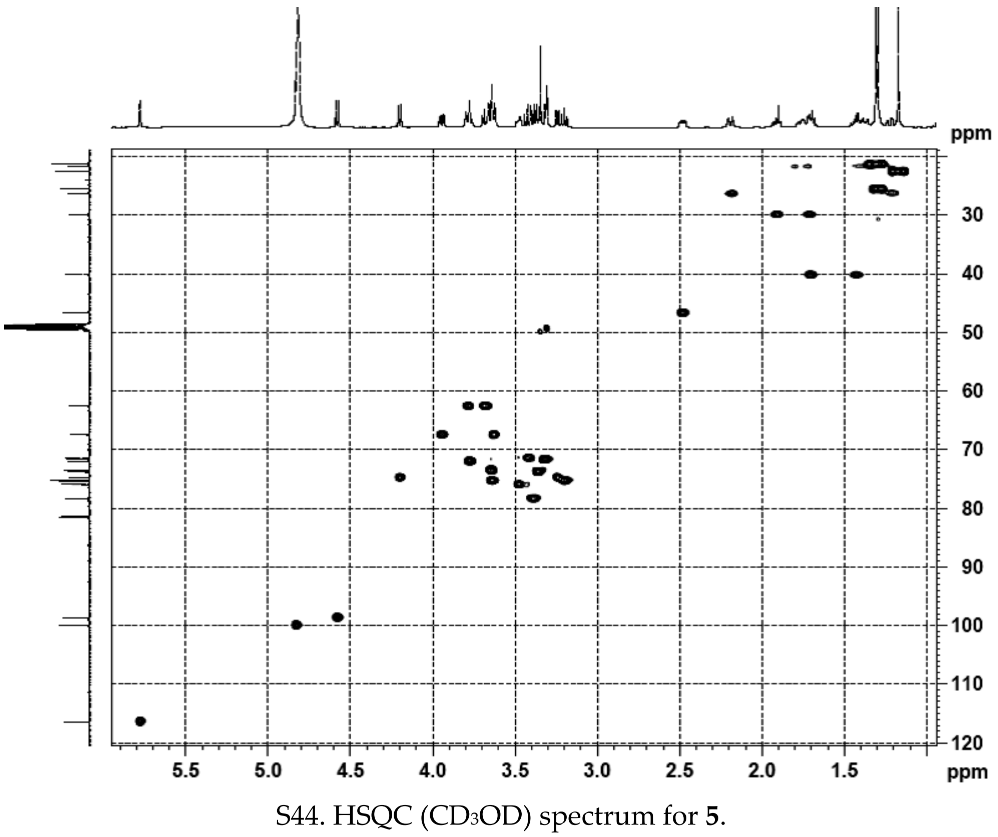

S44. HSQC (CD_3OD) spectrum for **5**.

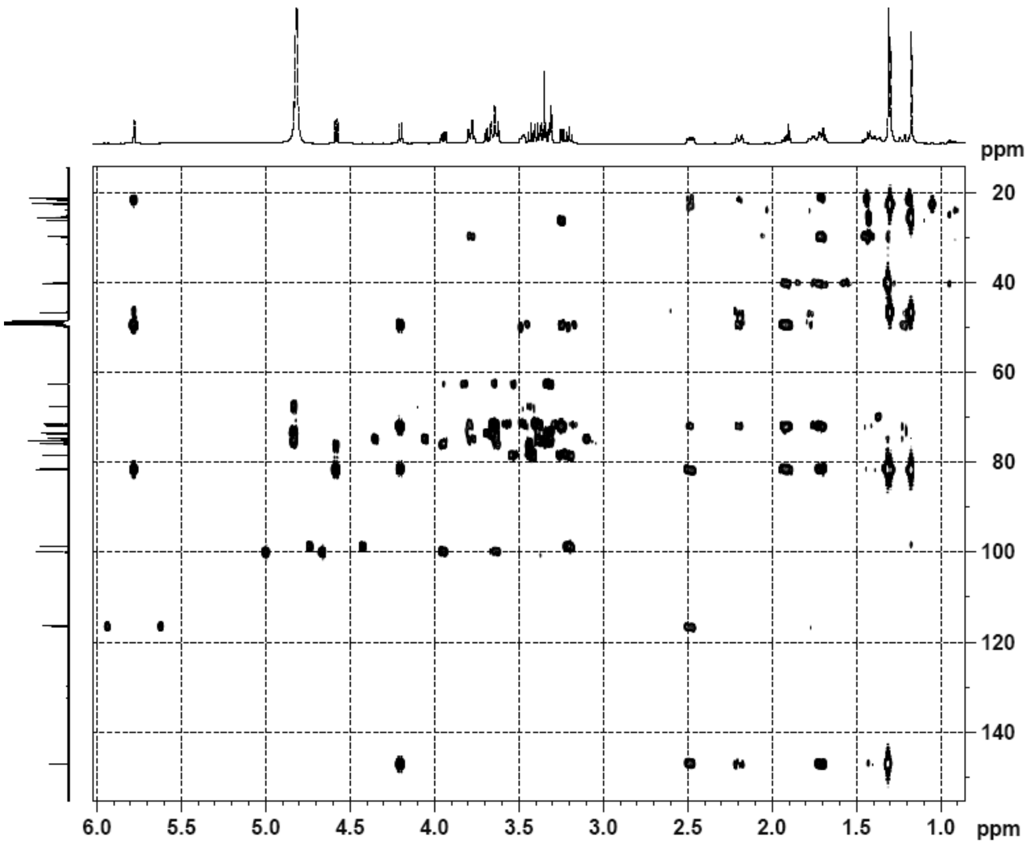

S45. HMBC (CD_3OD) spectrum for **5**.

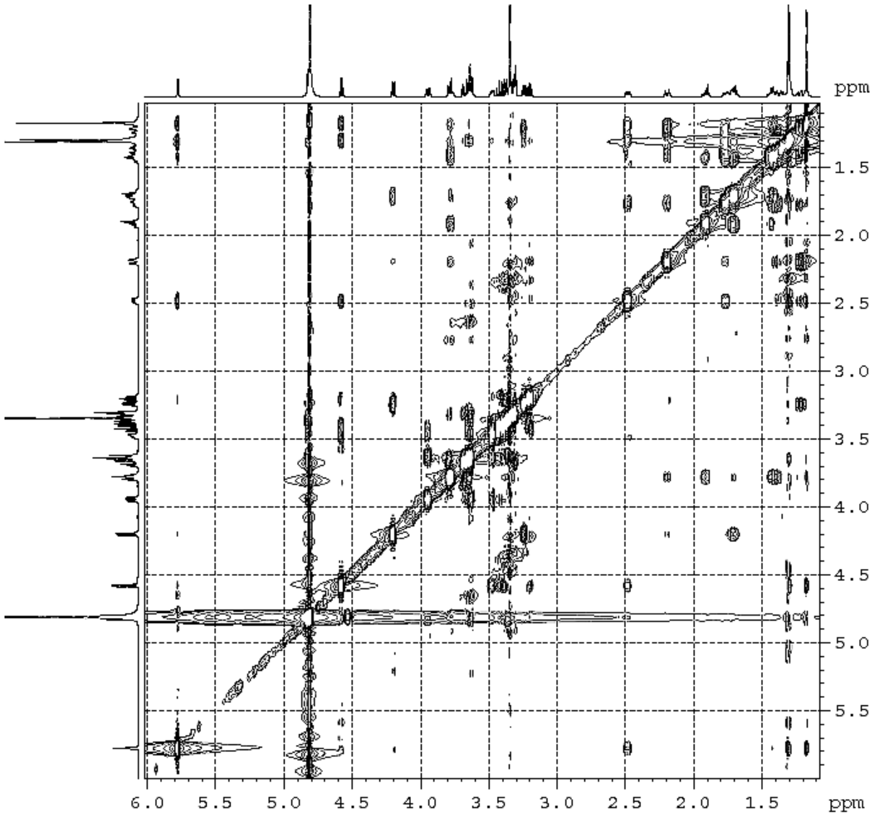

S46. NOESY (CD_3OD) spectrum for **5**.

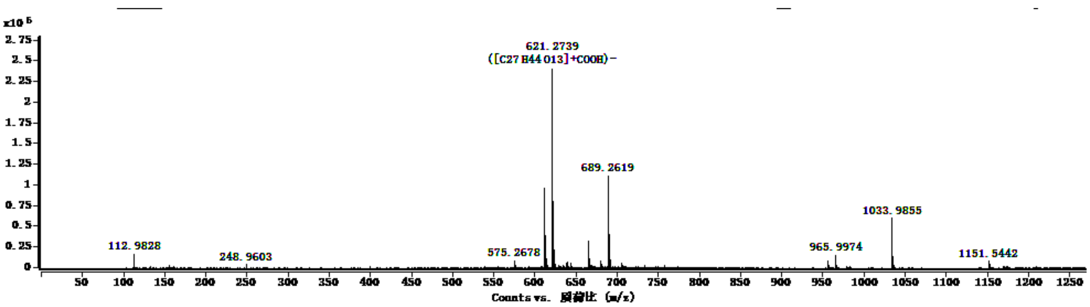

S47. HRESI-TOF-MS spectrum for **5**.

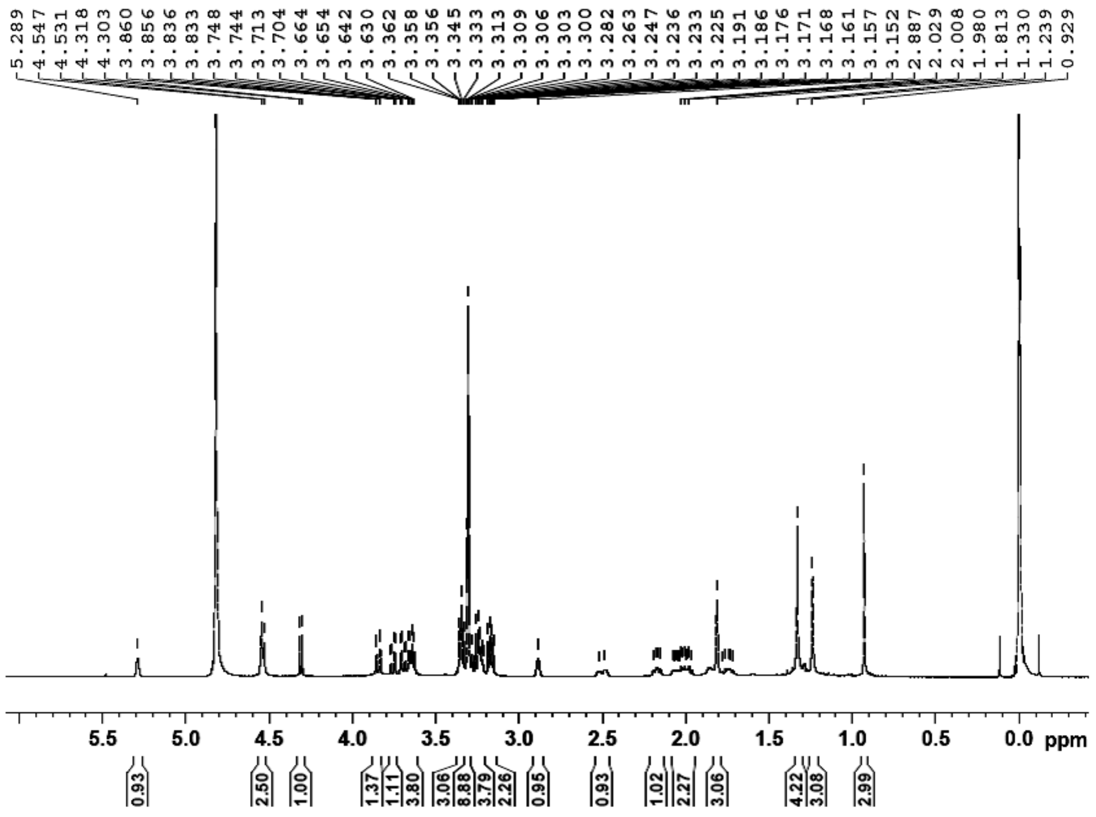

S48. ^{1}H NMR (500 MHz, CD_3OD) spectrum for **6**.

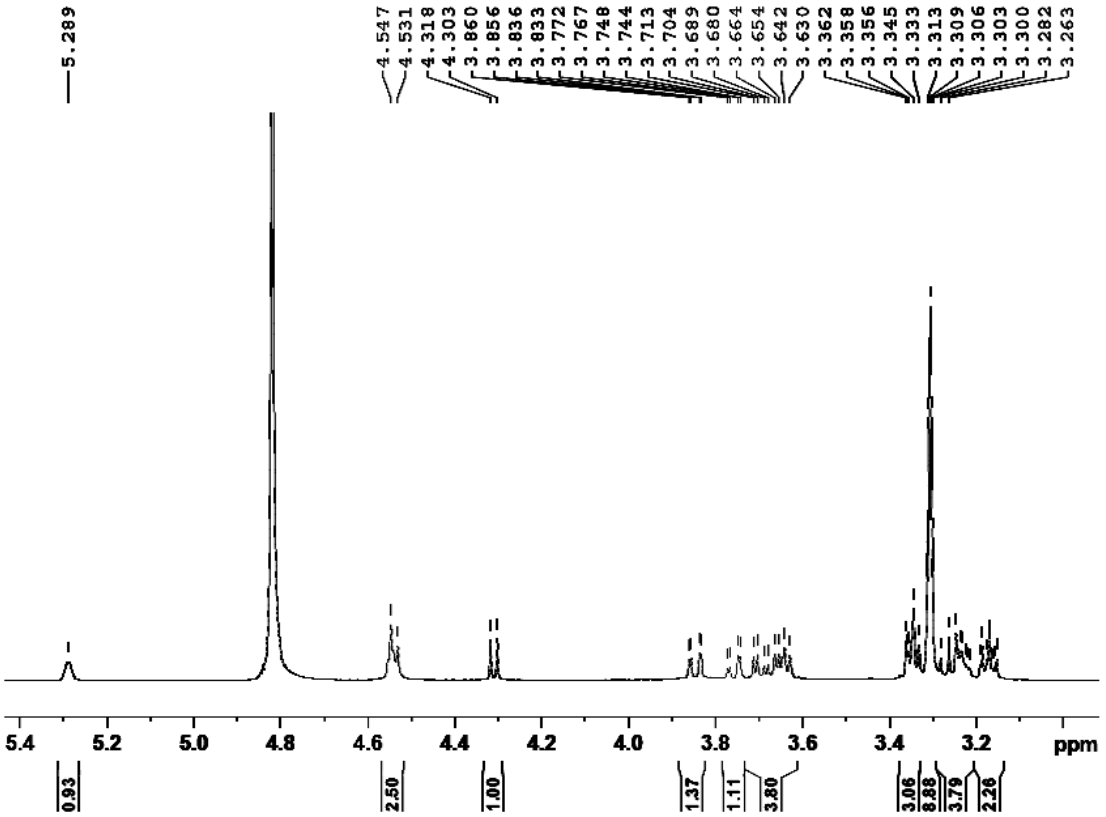

S49. Enlarged ^{1}H NMR (500 MHz, CD_3OD) spectrum 1 for **6**.

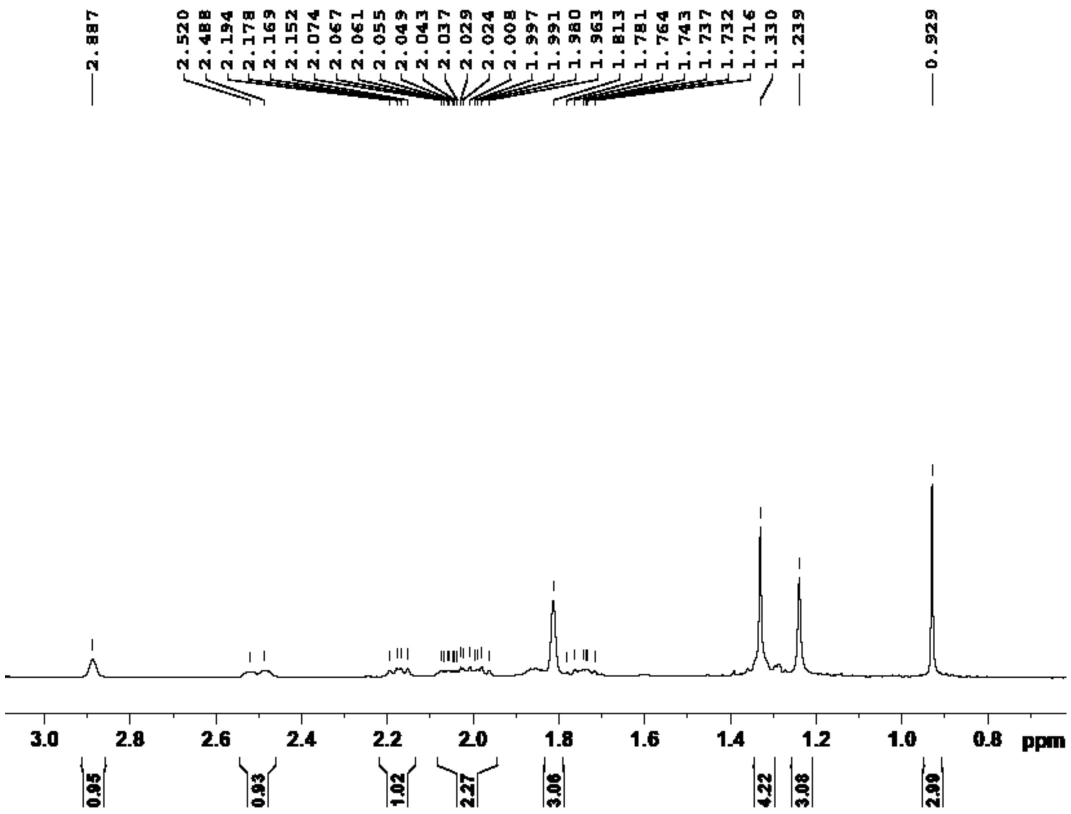

S50. Enlarged ^{1}H NMR (500 MHz, CD_3OD) spectrum 2 for **6**.

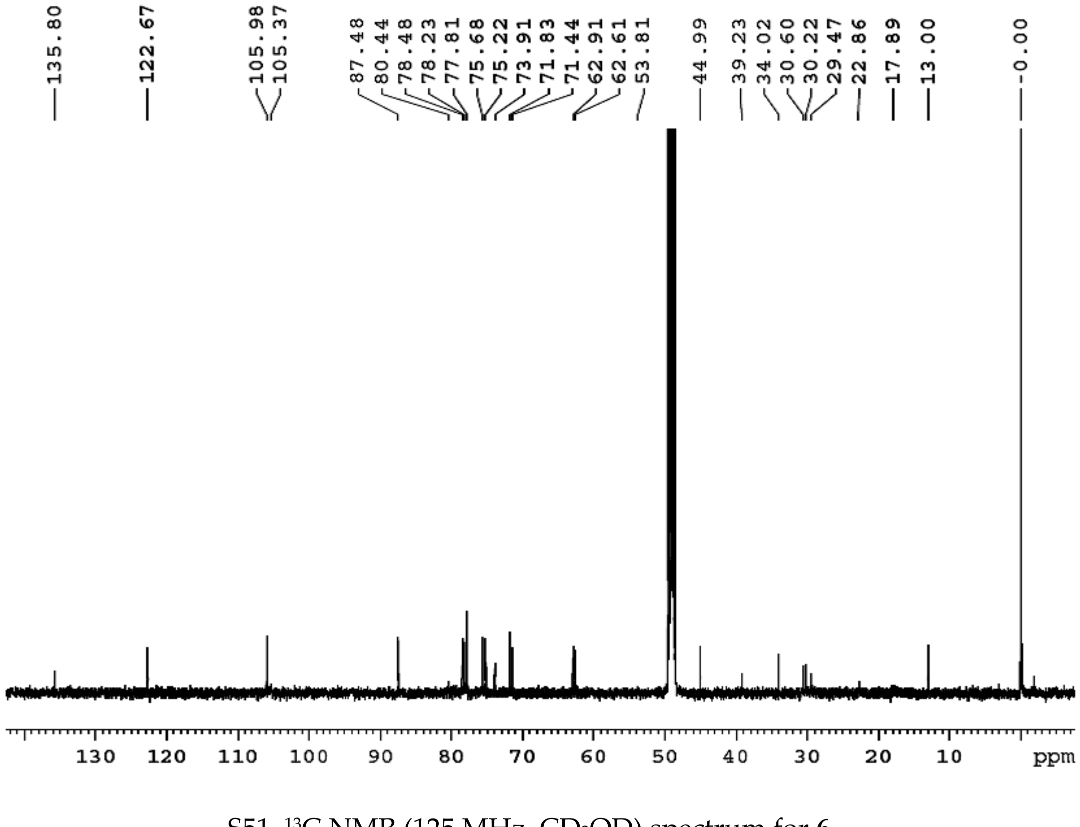

S51. ^{13}C NMR (125 MHz, CD_3OD) spectrum for **6**.

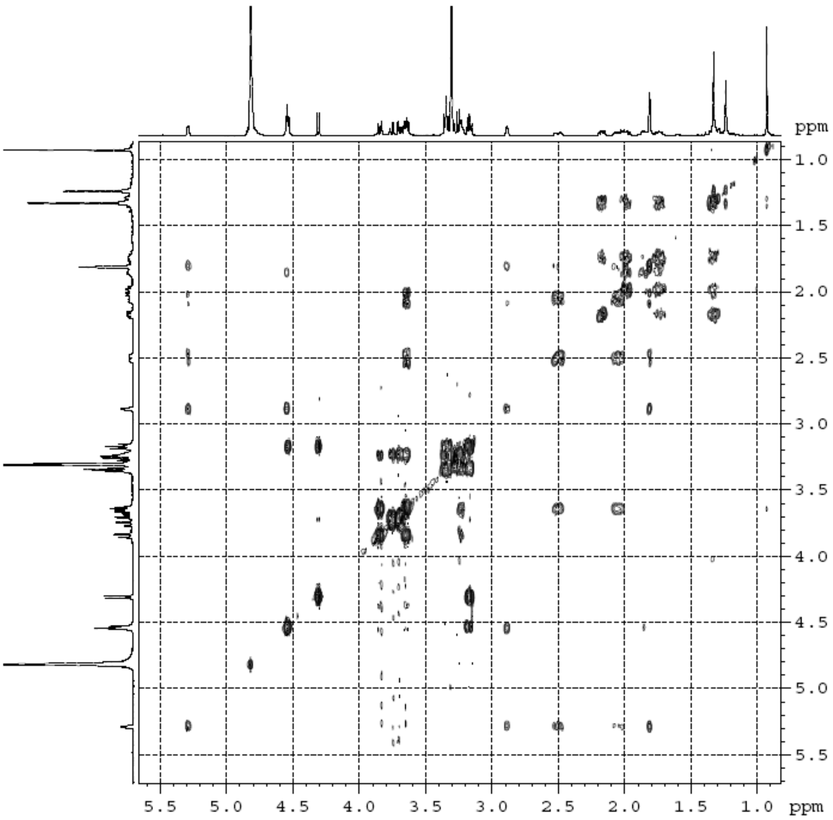

S52. ^{1}H ^{1}H COSY (CD_3OD) spectrum for **6**.

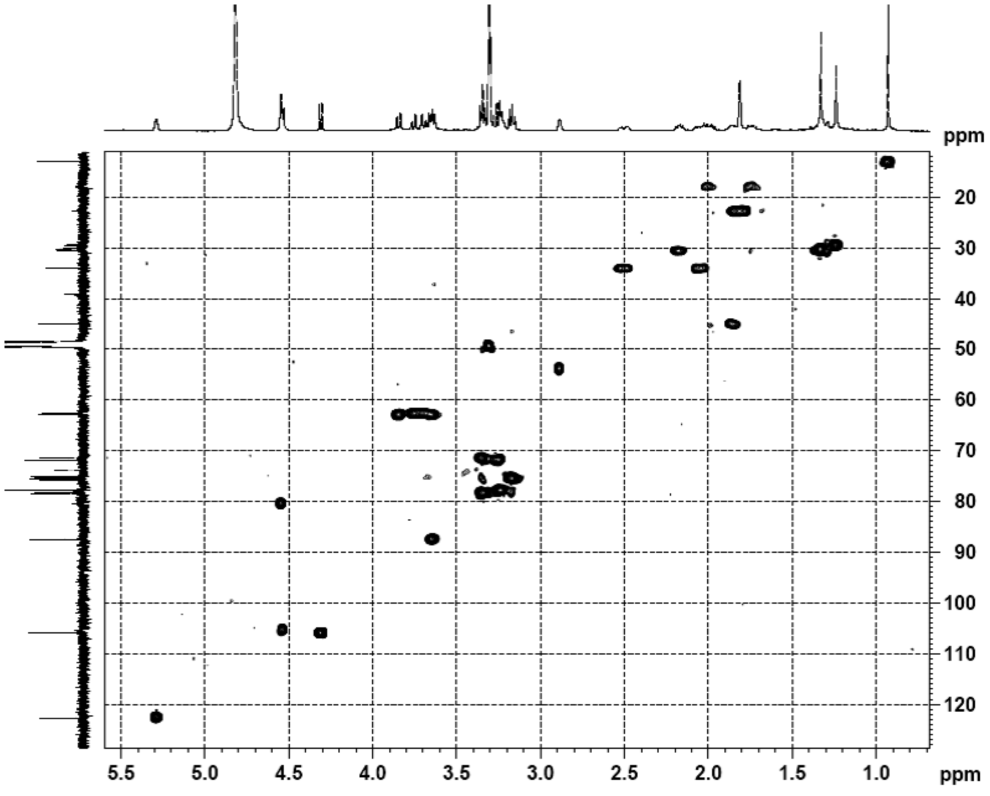

S53. HSQC (CD_3OD) spectrum for **6**.

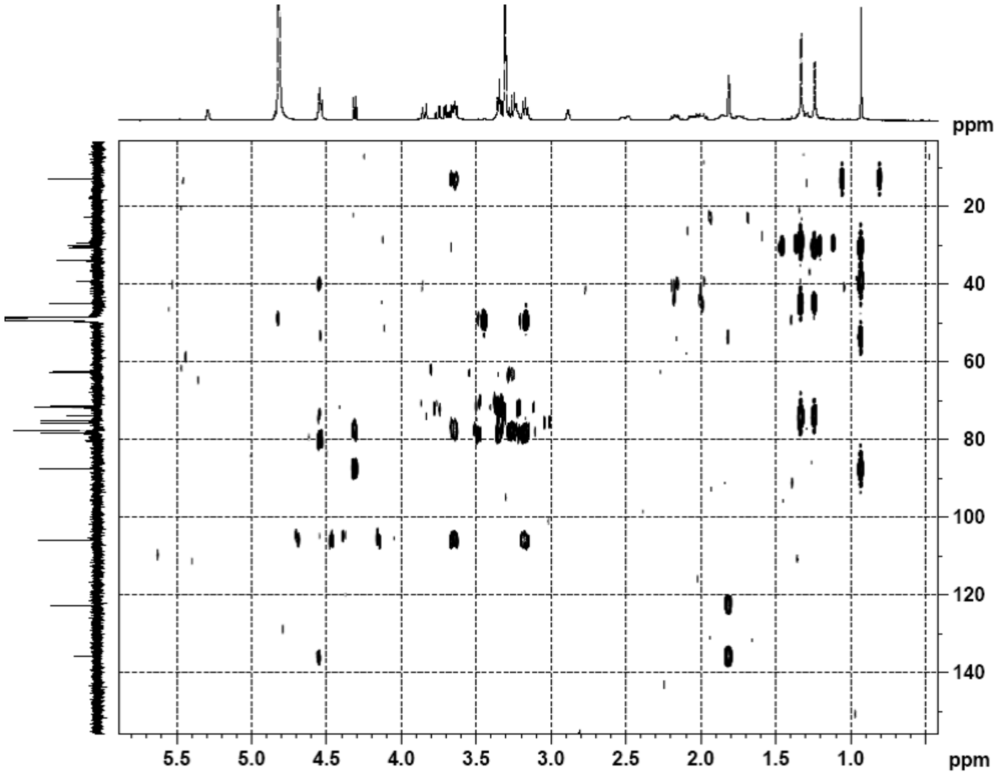

S54. HMBC (CD_3OD) spectrum for **6**.

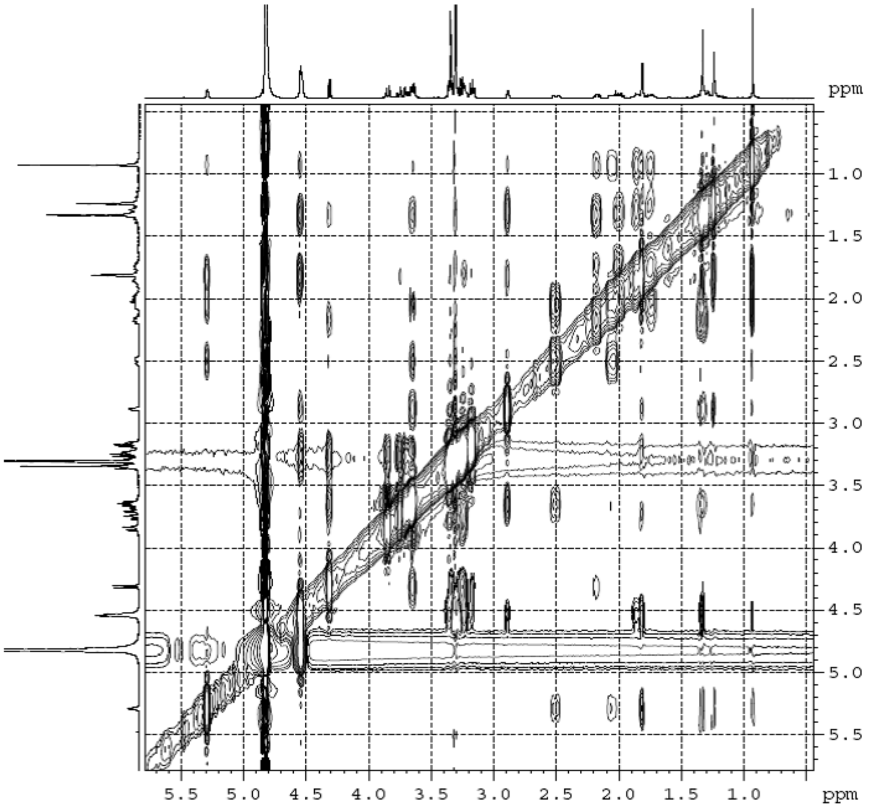

S55. NOESY (CD_3OD) spectrum for **6**.

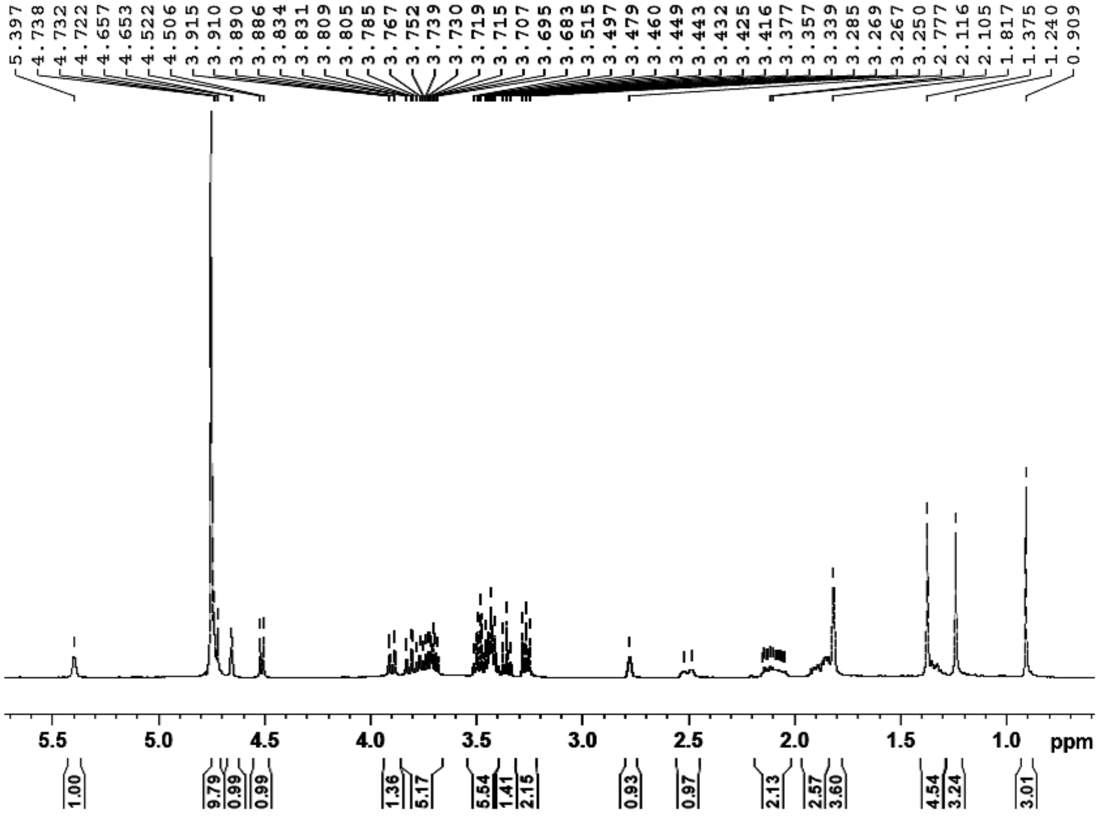

S56. ^{1}H NMR (500 MHz, D_2O) spectrum for **6**.

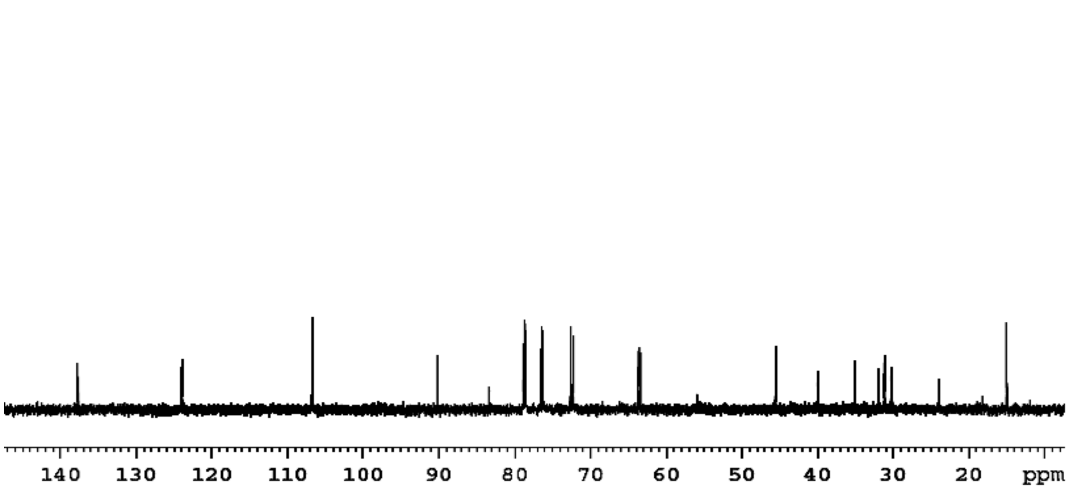

S57. [13]H NMR (125 MHz, D_2O) spectrum for **6**.

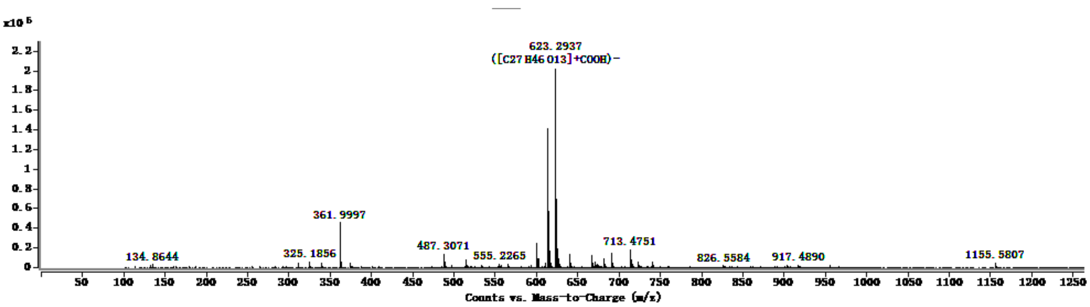

S58. HRESI-TOF-MS spectrum for **6**.
